# *Trypanosoma brucei* ribonuclease H2A is an essential R-loop processing enzyme whose loss causes DNA damage during transcription initiation and antigenic variation

**DOI:** 10.1093/nar/gkz644

**Published:** 2019-07-27

**Authors:** Emma Briggs, Kathryn Crouch, Leandro Lemgruber, Graham Hamilton, Craig Lapsley, Richard McCulloch

**Affiliations:** 1 The Wellcome Centre for Integrative Parasitology, University of Glasgow, College of Medical, Veterinary and Life Sciences, Institute of Infection, Immunity and Inflammation, Sir Graeme Davies Building, 120 University Place, Glasgow G12 8TA, UK; 2 Glasgow Polyomics, University of Glasgow, Wolfson Wohl Cancer Research Centre, Garscube Estate, Switchback Rd, Bearsden G61 1QH, UK

## Abstract

Ribonucleotides represent a threat to DNA genome stability and transmission. Two types of Ribonuclease H (RNase H) excise ribonucleotides when they form part of the DNA strand, or hydrolyse RNA when it base-pairs with DNA in structures termed R-loops. Loss of either RNase H is lethal in mammals, whereas yeast survives the absence of both enzymes. RNase H1 loss is tolerated by the parasite *Trypanosoma brucei* but no work has examined the function of RNase H2. Here we show that loss of *T. brucei* RNase H2 (TbRH2A) leads to growth and cell cycle arrest that is concomitant with accumulation of nuclear damage at sites of RNA polymerase (Pol) II transcription initiation, revealing a novel and critical role for RNase H2. Differential gene expression analysis reveals limited overall changes in RNA levels for RNA Pol II genes after TbRH2A loss, but increased perturbation of nucleotide metabolic genes. Finally, we show that TbRH2A loss causes R-loop and DNA damage accumulation in telomeric RNA Pol I transcription sites, also leading to altered gene expression. Thus, we demonstrate separation of function between two nuclear *T. brucei* RNase H enzymes during RNA Pol II transcription, but overlap in function during RNA Pol I-mediated gene expression during host immune evasion.

## INTRODUCTION

Incorporation of ribonucleotides is a major threat to the stability of DNA genomes. Such incorporation can occur in three ways, each of which can be tackled by ribonuclease H (RNase H) enzymes. Ribonucleotide monophosphates (rNMPs) can be directly incorporated into DNA by DNA polymerases (Pols), an error that occurs at various frequencies depending on the selectivity of dNTPs over rNTPs by the different types of DNA Pol and by the base type (1–3). The ratio of rNTPs/dNTPs also influences rNMP selection, with rNTPs exceeding dNTPs in the cellular pool (4). These factors result in as many as 13 000 and 3 million rNMPs being incorporated into the yeast and human genomes per round of replication, respectively (1,5,6). Once incorporated, ribonucleotides destabilise DNA due to the presence of a reactive 2′-hydroxyl group on the ribose sugar, rendering the DNA backbone more vulnerable to cleavage. rNMPs are further incorporated as RNA primers necessary for the initiation steps of DNA replication. Whereas leading strand replication initiates from a single origin and a single RNA primer, lagging strand replication requires 7–14 nucleotide RNA primers for the synthesis of each Okazaki fragment, which is normally ∼200 bp in length (7–9), meaning ribonucleotides are found through this DNA strand. RNA is also frequently found associated with genomic DNA in the form of R-loops: for instance, nascent RNA can become hybridised to the template DNA strand behind a transcribing RNA Pol, forming a heteroduplex and displacing a single strand of DNA (10). Alternatively, R-loops can form in *trans* when RNA generated at one genomic location forms an RNA–DNA hybrid elsewhere in the genome (11–13). Though R-loops have been linked to several genomic functions (10,14,15), including transcription, DNA replication, chromosome segregation and telomere homeostasis, the RNA–DNA hybrids can also lead to instability and mutation (16–18), particularly when RNA biogenesis is compromised (19–23) and at sites of clashes between the DNA replication and transcription machineries, potentially contributing to replication fork collapse (24,25).

All organisms encode RNase H enzymes that degrade RNA incorporated in DNA (26). Though RNase H enzymes can contribute to the removal of DNA replication-associated RNA primers, two other nucleases, flap endonuclease 1 (FEN1) and Dna2, appear to play a larger role in ensuring these ribonucleotides remain only transiently in DNA (7,27–29). In contrast, RNase H enzymes play a more critical role in removing embedded ribonucleotides and R-loops. Most organisms encode two RNase H enzymes, type 1 and type 2. Eukaryotic type 2 RNase H is termed RNase H2 and is a complex made up of catalytic subunit, A, and two further subunits, B and C. In contrast, RNase H1, the type 1 enzyme, is a monomer. Only RNase H2 is able to remove embedded ribonucleotides, which it does by initiation of the ribonucleotide excision repair (RER) pathway (30,31). In this reaction, RNase H2 detects the 2′-OH group and cleaves 5′ of an embedded ribonucleotide, resulting in a DNA nick. DNA Pol δ subsequently performs PCNA-dependent nick translation and displaces the ribonucleotide, which is then removed by FEN1. Finally, DNA ligase repairs the lesion. In contrast to the specific role of RNase H2 in RER, both eukaryotic RNase H enzymes are able to resolve R-loops (32), which they do by hydrolysing the RNA within the RNA–DNA hybrid. In yeast, both the RER and R-loop activities of RNase H2 are known to protect against genomic instability (33,34), although the protein is not essential for cell viability, even when gene mutation is combined with loss of RNase H1 (34). In contrast, RNase H1 and RNase H2 are both essential for mouse embryonic development: lack of the former impairs mitochondrial DNA replication, while lack of the latter results in increased levels of ribonucleotides and DNA lesions in the nuclear genome. In addition, in humans, mutations in all three RNase H2 subunits have been shown to cause the auto-inflammatory disease Aicardi–Goutières syndrome (AGS) (35). Mice lacking fully functional RNase H2 display features of AGS that result from aberrant activation of an innate immune pathway that normally targets foreign cytosolic DNA (36,37). However, what self-molecules cause aberrant autoimmunity in these AGS models, how they reach the cytosol, and what activity of RNase H2 causes their generation, remains unclear (38).

In previous work, we examined the distribution of R-loops across the genome of *Trypanosoma brucei*, comparing mammal-infective wild type (WT) cells with mutants lacking *T. brucei* RNase H1 (TbRH1). The *T. brucei* genome, in common with all kinetoplastids (39), is arranged radically differently from most eukaryotes, since virtually all protein-coding genes (∼8,000) are expressed by RNA Pol II from a relatively small number of multigenic transcription units, meaning each gene does not have its own defined promoter, but many (sometimes hundreds) of genes share a transcription start site, where conserved RNA Pol II promoter sequences have not been found. Genes within a single polycistronic transcription unit (PTU) are initially encoded as a potentially multigene pre-mRNA before mature mRNAs are generated by coupled 5′ RNA *trans*-splicing (adding the cap) and polyadenylation (40). RNA Pol II transcription initiation, as well as termination, has been mapped to so called strand switch regions (SSRs), which separate adjacent PTUs, including by RNA-seq (41) and chromatin immunoprecipitation (ChIP) of modified and variant histones (42), modified base J (43), and a subunit of RNA Pol II (44). Transcription in *T. brucei* also appears functionally linked with DNA replication, since at least one component of the origin recognition complex (ORC) binds to SSRs, although only a subset are activated during S phase (45,46). We have shown that the most abundant site of R-loop accumulation in the constitutively transcribed *T. brucei* ‘core’ genome is at intergenic sequences within the RNA pol II PTUs, where the RNA–DNA hybrids display precise association with regions of low nucleosome density, suggesting a relationship with polyadenylation and, perhaps, *trans*-splicing (47). R-loops are also notably enriched at SSR boundaries where RNA Pol II transcription initiates, whereas little or no R-loop signal is observed at SSRs where transcription termination takes place. Although R-loops increase at various loci after deletion of TbRH1 (47), no cell cycle or growth defects are observed, even though it might be predicted that R-loops in the PTUs present an obstacle to replication and transcription, or that they might form at potentially predictable sites of clashes between the *T. brucei* replication and transcription machineries, perhaps suggesting TbRH1-independent mechanisms to avoid such conflict.

Gene expression in *T. brucei* displays further novelty in that some proteins are transcribed by RNA Pol I, not II. Whilst resident in the mammalian host, trypanosomes express a dense ‘coat’ of variant surface protein (VSG), from one of ∼15 telomeric multigene RNA Pol I VSG bloodstream form expression sites (BES) (48). Co-transcribed with the VSG are multiple expression site-associated genes (ESAGs), most of which also encode surface proteins (49). In order to evade host immunity *T. brucei* continually switches between expression of antigenically distinct VSGs, a process termed antigenic variation (50). One mechanism for VSG switching is silencing transcription from the single active BES and activating transcription from a previously silent BES, containing a different VSG. Additionally, recombination mechanisms allow VSG sequences (from ∼2,000 genes and pseudogenes) to be copied from silent subtelomeric arrays, minichromosomes or the silent BES into the active BES (50,51). R-loops are found at low levels in the active BES of WT *T. brucei*, suggesting they form co-transcriptionally (52). Upon deletion of *TbRH1*, R-loop signal significantly increases in both active and silent BES, and is associated with the accumulation of DNA damage in the active BES and increased levels of expression of previously silent VSGs (52). Though these data implicate R-loops targeted by TbRH1 in VSG switching, how the RNA–DNA hybrids are linked to transcriptional and recombinational switching, and whether they are solely recognised by TbRH1 is unknown. For instance, increased DNA damage is mainly detected in S and G2 phase TbRH1 mutant parasites, but if and how DNA replication converts R-loops into BES DNA breaks is unknown (46,53). Moreover, overexpression of TbRH1 has been shown to decrease VSG switching in TbRAP1-depleted cells (54), but how such a telomere function relates to VSG transcription and recombination is unclear.

Here, we describe the function of RNase H2 in *T. brucei*, which we examined to attempt to clarify how R-loop formation and resolution contributes to RNA Pol II core transcription and to VSG transcription and recombination. We show that loss of the *T. brucei* RNase H2A catalytic subunit, TbRH2A, is lethal and leads to cell cycle stalling associated with extensive nuclear DNA damage, but without loss of DNA synthesis. Mapping reveals that DNA damage accumulates specifically at transcription initiation sites after loss of TbRH2A, where we also detect changes in R-loop distribution. Loss of TbRH2A also causes R-loop and DNA damage accumulation across the VSG ES, with increased changes in VSG expression. Finally, RNA-seq details differential gene expression of both RNA Pol I and II transcribed genes after TbRH2A loss. Thus, we demonstrate a separation of function between the two nuclear *T. brucei* RNase H enzymes in the context of multigene RNA Pol II transcription, but overlap in functions during antigenic variation.

## MATERIALS AND METHODS

### *T. brucei* cell line generation and culture

C-terminal endogenous epitope tagging was carried out by cloning 604 bp of the 3′ *TbRH2A* sequence, PCR-amplified using primers CGACGAAGCTTGAACACGCTTAGCCATCAAA*C* and GACGTCTAGAAGGGACTTCCCGCGACAAA, into a version of the pNAT plasmid containing six copies of the HA sequence (55). The construct was linearized by digestion with ApaI, before stable transfection into bloodstream form (BSF) *T. b. brucei* strain Lister 427 MITat1.2, where the construct was integrated immediately downstream of the *TbRH2A* coding sequence. Inducible RNAi targeting TbRH2A was accomplished using a genetically modified strain derived from Lister 427 MITat1.2, named 2T1 (56). Two inverted copies of 432 bp of the *TbRH2A* ORF, PCR-amplified using primers GGGGACAAGTTTGTACAAAAAAGCAGGCTCCGCAGCTATGACAGGTGTA and GGGGACCACTTTGTACAAGAAAGCTGGGTCTCGAAGACGATAGGGATG, were cloned into the pGL2084 vector (57) using Gateway BP Clonase. The construct was then linearised and transfected into the parental 2T1. Incubation of the resulting TbRH2A^RNAi^ cell line with 1 μg.ml^−1^ tetracycline induced transcription of the *TbRH2A*-specific hairpin RNA molecules, triggering RNAi. Lister 427 and 2T1 cells, as well as transformants derived from each, were maintained in HMI-9 (HMI-9 (Gibco), 20% (v/v) FCS (Gibco), Pen/Strep (Sigma) (penicillin 20 U/ml, streptomycin 20 μg/ml)), and HMI-11 (HMI-9 (Gibco), 10% (v/v) FCS (Gibco, tet free), Pen/Strep (Sigma) (penicillin 20 U/ml, streptomycin 20 μg/ml)) media, respectively. During EdU uptake assays, cells were instead incubated in thymidine-free HMI-11 media (Iscove's modified Dulbecco's medium (IMDM) (Gibco), 10% (v/v) FBS (Gibco, tet free), 1% (v/v) Pen/Strep (Gibco), 4% (v/v) ‘HMI-9 mix’ (0.05 mM bathocuproine disulphonic acid, 1 mM sodium pyruvate and 1.5 mM l-cysteine) (Sigma Aldrich), 1 mM hypoxanthine (Sigma Aldrich) and 0.0014% 2-mercaptoethanol (Sigma Aldrich)). In all cases, 2T1 cells lines were grown in the presence of 0.5 μg.ml^−1^ puromycin (InvitroGen) and 2.5 μg.ml^−1^ phleomycin (InvitroGen), and TbRH2A^RNAi^ parasites were cultured with 2.5 μg.ml^−1^ phleomycin (Invitrogen) and 5 μg.ml^−1^ hygromycin B (Calbiochem) to maintain the RNAi construct.

### Fluorescent-activated cell sorting (FACS)

∼3 × 10^6^ cells were collected per sample and fixed with 1% formaldehyde (FA) for 10 min at room temperature before being permeabilized with 0.01% Triton X-100 for 30 min on ice. Cells were incubated with 100 μg.ml^−1^ RNase A and 15 μg.ml^−1^ propidium iodide (PI) for 30 min before PI was detected using the BD FACSCalibur™ (BD Biosciences). Data was analysed using FlowJo_V10™ software (FlowJo, LLC).

### Immunofluorescence imaging

All immunofluorescence assays were performed as previously described (52). For detection of epitope-tagged TbRH2A and γH2A, cells were fixed with 4% FA for 4 min before quenching with 100 nM glycine and permeabilization with 0.2% Triton X-100 for 10 min. 3% bovine serum albumin was using to block samples before staining with primary (α-myc Alexa Fluor 488 conjugated (Millipore), 1:500; α-γH2A, 1:1000) then secondary antibodies (Alexa Fluor 488 goat α-rabbit (Molecular Probes), 1:1000) and mounting in Fluoromount G with DAPI (Cambridge Bioscience, Southern Biotech). For VSG immunofluorescence cells were fixed in 1% FA and blocked in 50% foetal calf serum, before staining with primary (α-VSG221, 1:10 000; α-VSG121, 1:10 000: gift from D. Horn) and secondary (Alexa Fluor 594 goat α-rabbit (Molecular Probes) 1:1000; Alexa Fluor 488 goat anti-rat (Molecular Probes), 1:1000) antibodies and DAPI mounting as above. Fluorescent imaging was performed with an Axioscope 2 fluorescence microscope (Zeiss) and a Zeiss PlanApochromat 63×/1.40 oil objective. High-resolution images were taken using an Olympus IX71 DeltaVision Core System microscope (Applied Precision) and SoftWoRx Suite v2.0 (Applied Precision) software. Either an Olympus PlanApo 60×/1.42 or an UplanSApo 100×/1.40 oil objective was used. Z-stacks of 5–6 μm thickness were acquired in 25 sections, then deconvolved with the ‘conservative’ method and high noise filtering. Fiji software was then used to generate maximum projection images. Super-resolution structured illumination microscopy (SR-SIM) was performed with an ELYRA PS.1 Microscope (Zeiss), using a Plan-Apochromat 63×/1.40 Oil DIC objective and 405, 488 and 594 nm lasers. Z-stack sections were ∼0.15 μm in thickness and totalled ∼10 μm. Image reconstruction was performed with ZEN Black software (Ziess) and 3D rendering with Imaris software (Bitplane) to produced 3D models.

### Western blot

Whole cell protein extracts were harvested from ∼ 2.5 × 10^6^ cells per sample by boiling in 10 μl loading buffer (1× NuPAGE^®^ LDS Sample Buffer (Life Technologies), 0.1% β-mecaptoletanol) for 10 min. Proteins were separated using NuPAGE Novex^®^ 10–12% Bis-Tris protein gels (Life Technologies) and transferred to PVDF membranes. Proteins were detected with anti-γH2A (1:1000) and mouse anti-Ef1α clone CBP-KK1 (1:25 000; Millipore) primary antibodies, and goat anti-mouse/rabbit IgG (H+L) horseradish peroxidase conjugates (1:5000; Life Technologies).

### EdU incorporation assays

Cells were incubated with 150 μM 5-ethynyl-2′-deoxyuridine (EdU) for 4 h prior to fixation at each time point with 1% FA at room temperature for 10 min, then permeabilized in 0.5% Triton X-100 for 20 min. EdU was detected by incubation for 1 h with the follow Click-It reaction mix: 21.5 μl 1× Reaction Buffer, 1 μl CuSO4, 0.25 μl Alexa Fluor 555 Azide and 2.5 μl 1× Additive Buffer. For dual staining for γH2A, cells were washed before incubating with anti-γH2A antisera (1:1000) then anti-rabbit Alexa Fluor 594 (1:1000), both in 3% BSA, and mounted in Fluoromount G with DAPI.

### DRIP/ChIP analysis

Immunoprecipitation of both RNA–DNA hybrids (DRIP) and γH2A was performed using the ChIP-IT Enzymatic Express kit (Active Motif) with formaldehyde fixed chromatin samples, using the hybrid-targeting S9.6 (4.5 ng, Kerafast) and α-γH2A (10 μl, homemade) antibodies respectively. Both were carried out as described previously (47). DRIP-ChIP-qPCR analysis was carried out as described previously (52) with on-bead *Escherichia coli* RNase HI treatment of DRIP samples. Target sequences were amplified with the following primers: VSG221, AGCAGCCAAGAGGTAACAGC and CAACTGCAGCTTGCAAGGAA; VSG121, AGGAAGGCAAATACGACCAG and TTGGGGTAAAAGTCCTTG; ESAG6, TGGGAGGGATGGATGTAATTT and CCGACCCCCCTTCCAAT; ESGA8, CGGATGCGTCGTGGAA and CCTCCGATACGCCGTTGA.

DNA libraries were prepared using the TruSeq ChIP Library Preparation Kit (Illumina). Fragments of 300 bp, including adaptors, were selected with Agencourt AMPure XP (Beckman Coulter) and sequenced using the Illumina NextSeq 500 platform. Analysis of DRIP-seq and γH2A ChIP-seq data was carried out as previously published (47). To allow downstream analysis to focus on the γH2A signal specific to the TbRH2A depletion induced DNA damage, the ratio of read enrichment was calculated for tet-induced samples relative to un-induced sample coverage (having first normalized to the respective input sample) for both 24 and 36 h timepoint data sets. All normalization and metaplot analysis was performed with the deepTools software suite (58).

### RNA-seq analysis

Total RNA was extracted, in duplicate, from 1 × 10^7^ cells using the RNeasy Mini Kit (Qiagen) and the TruSeq Stranded mRNA kit (Illumina) was used to prepare poly(A) selected libraries of ∼300 bp fragments. Sequencing was performed with the NextSeq 500 platform giving paired-end reads of 75 bp. Mapping was performed with HiSat2 v2.0.5 using default parameters with the exception of not permitting splice alignments (–no- splice-alignment), to either a ‘hybrid’ reference genome, consisting of the 11 Mb chromosome assemblies of the *T. brucei* TREU927 v5.1 genome, 14 BES contigs and 5 mVSG expression site (ES) contigs (59), or a collection of 2470 VSG coding regions of the *T. brucei* Lister427 strain (60). Reads with MAPQ score <1 were removed before counting with HTseq-count software using default parameters. Normalization and differential expression was carried out with DESeq2 v1.18.1 (61), considering data from 24 and 36 hr time points separately. GO term analysis was performed using Cytoscape v3.6.1 (62) plugin BiNGO (63) with hypergeometric statistical testing of significance and multiple testing correction with the Benjamini and Hochberg False Discovery Rate (FDR) correction. FDR adjusted *P* values <0.01 were deemed significantly enriched terms.

## RESULTS

### RNase H2A is an essential nuclear protein in bloodstream form *T. brucei*

In order to identify putative type 2 RNase H proteins in *T. brucei*, BLAST and protein domain analyses were employed, searching the *T. brucei* genome with both type 1 and type 2 RNase H proteins from *E. coli* and a range of eukaryotes. As we (52) and others (64) have described, a single RNase H1 can be readily detected in *T. brucei* (TbRH1). In addition, three candidates for the *T. brucei* RNase H2 complex were revealed: Tb427.10.5070 was predicted to encode a protein highly similar to eukaryotic catalytic RNase H2A subunits, and Tb427.01.4220 and Tb427.01.4730 encode likely orthologues of RH2B and RH2C, respectively (Supplementary Table S1, Figure S1A). Surprisingly, synteny between these orthologues and three previously described RNase H2-like genes in *Leishmania major* (65) is not simple to discern. The predicted amino acid sequence of Tb427.10.5070 revealed conservation of active site and catalytic residues described in type 2 RNase H enzymes in other organisms (Supplementary Figure S1B), consistent with the gene encoding the catalytic subunit of RNase H2. To begin to test for function, TbRH2A was C-terminally tagged with 6 copies of the HA epitope and expressed from its endogenous locus in mammalian-infective (bloodstream form; BSF) parasites (Supplementary Figure S2A). Immunofluorescence with anti-HA antiserum revealed signal throughout the nucleus in all cell cycle stages and without any discernible sub-nuclear localisation (Supplementary Figure S2B, C), features shared with TbRH1 (52), suggesting the presence of two RNase H enzymes in the *T. brucei* nucleus. Also in common with TbRH1, TbRH2A-6HA fluorescence signal increased in cells undergoing nuclear DNA replication (Supplementary Figure S2D).

**Figure 1.**
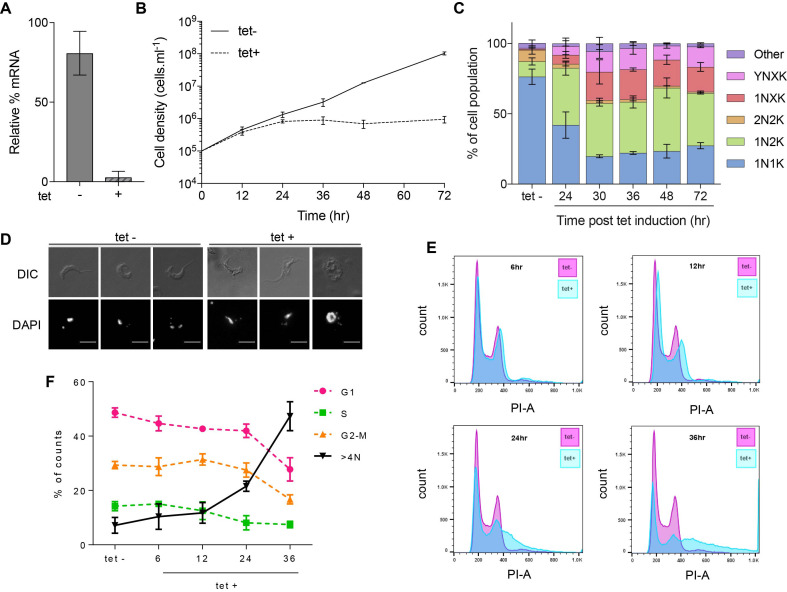
RNase H2A is essential for bloodstream form *T. brucei* viability. (**A**) Levels of *TbRH2A* transcripts in tetracycline (tet) induced (+) and uninduced (–) cells after 36 h of culture, relative to 2T1 cells (levels set at 100%), determined by RT-qPCR; error bars show SD of two independent experiments. (**B**) Cumulative growth curves of tet+ and tet- TbRH2A RNAi cultures, showing cell densities over 72 h (uninduced cells were diluted at 24 and 48 h). (**C**) Bar graph showing, at multiple time points, the percentage of tet-induced cells in the population that correspond to the following cell types, defined by DAPI staining of the nucleus (N) and kinetoplast (K): 1N1K, 1N2K, 2N2K, 1NXK (>2 K foci), YNXK (>2 K foci and aberrant N number or morphology), and other (do not conform to the above). Tet- shows the average of uninduced samples from all time-points. >200 cells were examined at each time point and error bars depict SD from three independent experiments. (**D**) Example images of induced (tet+) and un-induced (tet–) cells after 30 h of growth; scale bar, 5 μm. (**E**) Profiles of propidium iodide (PI) stained uninduced (tet–, pink) and RNAi induced (tet+, blue) populations after 6, 12, 24 and 36 h growth; y-axes show cell counts, and x-axes shows PI-area fluorescence. (**F**) Graph showing the percentage of cells in each expected cell cycle stage (G1, S and G2-M), or cells with genome content >4N, based on measuring proportion of the population with 2N, 2N-4N, 4N and >4N content; tet– shows the average of all tet– time points. In (B), (C) and (F), error bars show SD of three independent experiments.

Attempts to generate *TbRH2A* null mutants were unsuccessful (data not shown), suggesting the protein is essential. To test this prediction, we employed tetracycline (tet)-inducible RNA interference (RNAi) (57). *TbRH2A* transcript levels were reduced to ∼3% of the parental cells when TbRH2A^RNAi^ cells were cultured in the presence of tet for 36 h (Figure 1A). Indeed, *TbRH2A* RNA levels were ∼19% lower in TbRH2A^RNAi^ cells than in the parental cells even when grown in the absence of tet, indicating some expression of the RNAi-inducing *TbRH2A* stem-loop RNA prior to induction, consistent with some altered phenotypes prior to RNAi induction (described below). Nonetheless, tet-induction of RNAi caused a severe growth defect relative to uninduced cells, with cell proliferation stalling 24 h post-induction (Figure 1B). Cell cycle progression (as analysed by staining nuclear (n) and kinetoplast (k) DNA with DAPI) was also severely altered: after 24 h of RNAi induction, the proportion of cells with one nucleus and two kinetoplasts (1N2K) increased to ∼40% of the population (Figure 1C), representing a ∼4-fold increase compared with uninduced cells (where ∼10% were 1N2K) and suggesting a stall in nuclear G2/M phase. Small numbers of cells with one nucleus and more than two kinetoplasts (1NXK) could also be detected at 24 h, and increased substantially at 30 h induction, indicating kDNA replication continued after depletion of TbRH2A (Figure 1C, D). Similarly, the small numbers of cells (∼3%) seen after 24 h induction that had multiple nuclei or aberrant nuclear staining, as well as >2 kinetoplasts (YNXK), increased after 30 h of RNAi (Figure 1C, D). These complex perturbations in growth after loss of TbRH2A, suggestive of a partial cell cycle stall and some further, ineffective nuclear replication and division, appeared not to increase from 36–72 h (Figure 1C), consistent with the lack of population growth or death during this time (Figure 1B). To further investigate the effects of TbRH2A depletion on the cell cycle, flow cytometry was used to examine DNA content (Figure 1E, F). Reduction in cells with 2C content (G1 phase) was apparent, consistent with the loss of 1N1K cells in the DAPI staining. However, the proportion of cells with 4C content (G2/M) decreased until 36 hr of tet-induction (∼18.2%), which is inconsistent with the increased number of 1N2K cells seen by DAPI. An explanation most likely lies in the pronounced increase of cells detected with more than diploid genome content (>4N, from ∼7% at 0 h to ∼47% at 36 h), suggesting that many of the cells scored as 1N2K, and apparently stalled in the cell cycle at G2/M, continued to synthesise nuclear DNA but mainly failed to effectively execute mitosis.

### DNA synthesis continues in RNase H2A depleted *T. brucei* despite increased nuclear DNA damage

**Figure 2.**
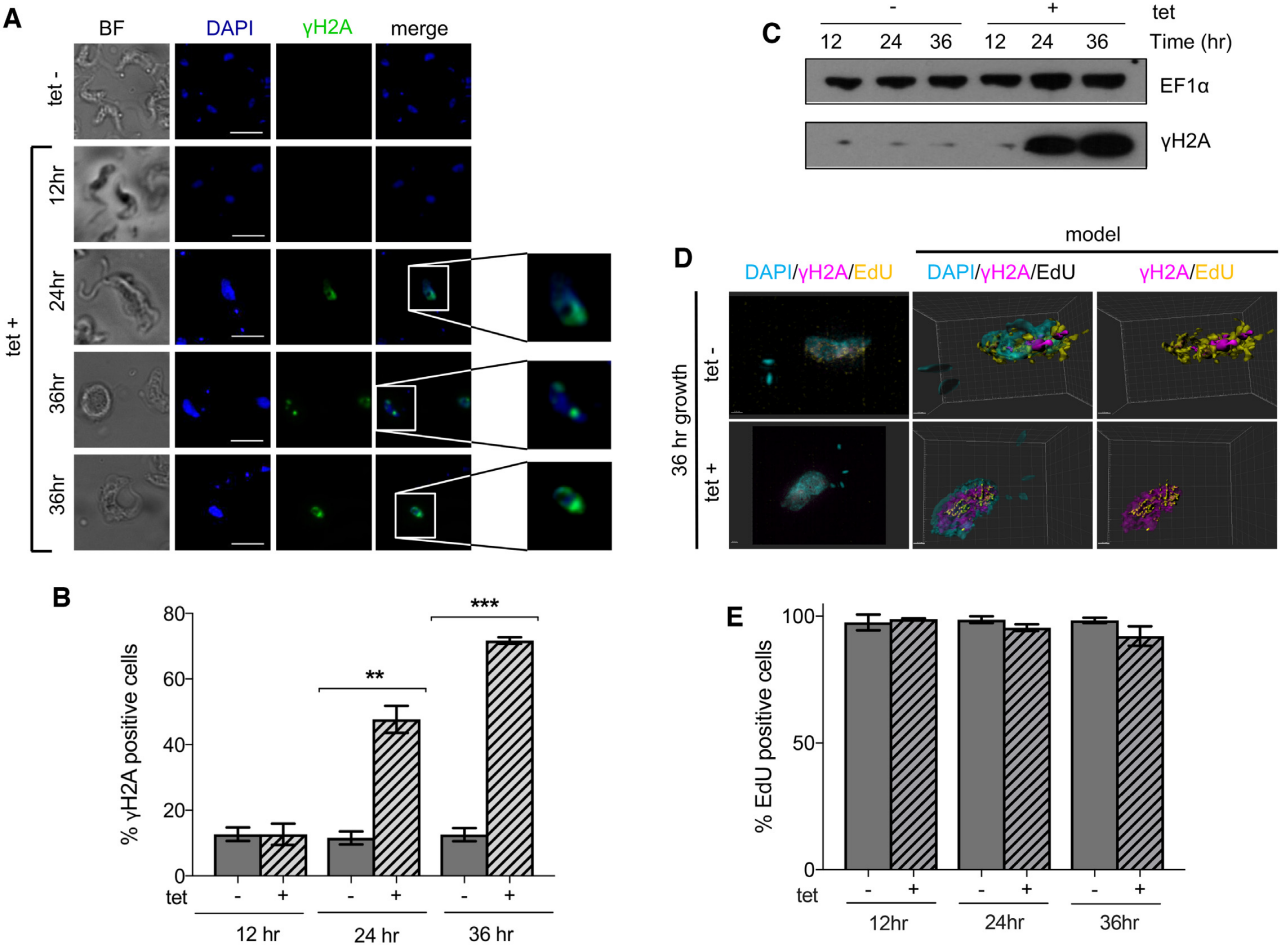
TbRH2A-depleted parasites accumulate DNA damage yet continue to synthesise DNA. (**A**) High-resolution imaging of DAPI (blue) and γH2A (green) immunofluorescence with (tet+) and without (tet–) RNAi induction, at various time points; scale bar, 5 μm. Images to the right show increased magnification of the boxed nuclear DNA. (**B**) Bar graphs showing the percentage of tet+ and tet– populations positively staining for γH2A after 12, 24 and 36 h growth; error bars show SD of three independent experiments. (**C**) Western blot detection of γH2A in whole cell protein extracts after 12, 24 and 36 h growth of tet+ and tet– cells; EF1α staining is shown as a loading control. (**D**) Example SR-SIM images of DAPI, γH2A and EdU staining are shown, along with 3D reconstructions (model). TbRH2A RNAi cells were cultured in the presence (tet+) or absence (tet–) of tet for 36 h before imaging; scale bar = 1 μm. (**E**) Bar graph showing the percentage of tet+ and tet– populations positively staining for EdU incorporation after 12, 24 and 36 h of growth; error bars show the SD for three independent experiments (further examples of immunofluorescence images of EdU and γH2A staining are shown in Supplementary Figure S3).

To ask if loss of TbRH2A affects nuclear genome functions, we measured levels of DNA damage before and after TbRH2A RNAi using antiserum recognising Thr130-phosphorylated histone H2A (γH2A), a known marker of DNA damage in *T. brucei* (66,67). Consistent with previous reports (66), immunofluorescence (IF) with anti-γH2A antiserum revealed nuclear signal in a small fraction (∼10%) of uninduced cells (Figure 2A, B). In contrast, IF of cells grown for 12, 24 and 36 h in the presence of tet (Figure 2A, B) revealed dramatic time-dependent increases in nuclear γH2A signal, reaching ∼75% of the RNAi induced cells in the population after 36 h (Figure 2B). Western blotting of whole cell protein extracts confirmed the IF data, with γH2A levels showing large increases 24 and 36 h after RNAi induction compared with uninduced cells (Figure 2C). To explore how such widespread nuclear damage relates to replication of the nuclear genome, we incubated RNAi induced and uninduced parasites with the thymine analogue 5-ethynyl-2′-deoxyuridine (EdU) and detected its incorporation via Click-IT chemistry (Figure 2D, E; Supplementary Figure S3). ∼99% of cells, cultured in the absence of tet, incorporated EdU in the nucleus after a 4 h incubation with the analogue (Figure 2E, Supplementary Figure S3), which is as expected for asynchronous BSF *T. brucei* cells with a cell cycle time of ∼6 h, since virtually all cells should, at least partially, undergo nuclear S phase and take up EdU. Even after 36 h of RNAi induction, when population growth had stopped (Figure 1B), ∼93% of cells incorporated EdU (Figure 2D, E; Supplementary Figure S3A) and we did not detect changes in EdU signal in individual cells after RNAi induction (Supplementary Figure S4). Hence, loss of TbRH2A had little, if any effect, on DNA synthesis. Moreover, since virtually all the RNAi-induced cells that had γH2A signal in their nucleus had also incorporated EdU, with overlap of the two signals (Figure 2D; Supplementary Figure S3), the extensive nuclear DNA damage caused by loss of TbRH2A did not appear to impede DNA replication, consistent with flow cytometry indicating increased DNA content in the absence of effective mitosis (Figure 1C, E, F).

### R-loop localisation at transcription start sites is altered after depletion of RNase H2A

To ask if the extensive nuclear genome damage seen after TbRH2A RNAi relates to R-loop distribution, we used monoclonal antibody S9.6 (68) to immunoprecipitate DNA-RNA hybrids from formaldehyde-fixed chromatin derived from RNAi-induced or uninduced cells, evaluating distribution by mapping reads to the *T. brucei* genome after next generation DNA sequencing (DRIP-seq). Supplementary Figure S5 shows genome-wide DRIP-seq mapping after 24 h of growth with and without tet-induced RNAi, revealing widespread R-loop enrichment and some correlation with DNA repeats. To understand how R-loop distribution in the TbRH2A^RNAi^ cells compared with similar analysis in *TbRH1* null mutants and WT *T. brucei* cells (47), DRIP-enriched regions were defined as locations with ≥1.2-fold-change increase in IP mapped reads relative to pre-IP samples. Analysis of these enriched regions showed they were mainly found in RNA Pol II PTUs (∼88% of uninduced, and ∼86% of RNAi induced; Supplementary Figure S6), a very similar distribution to that previously reported for WT and *TbRH1* null mutant DRIP-seq data. Moreover, DRIP enriched regions within the PTUs, before and after TbRH2A RNAi, were most clearly associated with intergenic sequences (∼58% of uninduced, and ∼60% of RNAi induced samples; Supplementary Figure S7A, B), a localization bias that appeared slightly increased compared with WT cells (∼50% of intra-PTU enriched regions; Supplementary Figure S7A). Correspondingly, the number of DRIP enriched regions associated with gene coding DNA sequence (CDS) was reduced in both the TbRH2A uninduced (6715 regions) and, even more so, in the RNAi induced (5300 regions) DRIP-seq data compared with WT (8861 regions; Supplementary Figure S7A). Heatmaps of DRIP-seq enrichment around every RNA Pol II gene confirmed the predominant enrichment around the CDS, with relatively precise signal localisation upstream and downstream of each CDS, as was seen in WT cells (Supplementary Figure S8) and *TbRH1* null mutants (47). Taken together, these data indicate relatively stable accumulation of R-loops within the RNA Pol II PTUs, which is not markedly altered by loss of TbRH2A or TbRH1 (47). Indeed, outside the RNA Pol II PTUs, DRIP-seq of both induced and uninduced TbRH2A^RNAi^ cells revealed R-loop enrichment in Pol I and Pol III transcribed genes, retrotransposon hotspot (RHS) genes, and in centromeres (Supplementary Figure S6), in each case at comparable levels to WT cells and TbRH1 null mutants (47), suggesting many of the R-loops that form in the *T. brucei* genome are relatively unaffected by loss of either RNase H activity. However, within this context of global R-loop stability, two regions displayed notable changes in DRIP-seq profile after TbRH2A loss: transcription start sites, and VSG genes (as described below).

**Figure 3.**
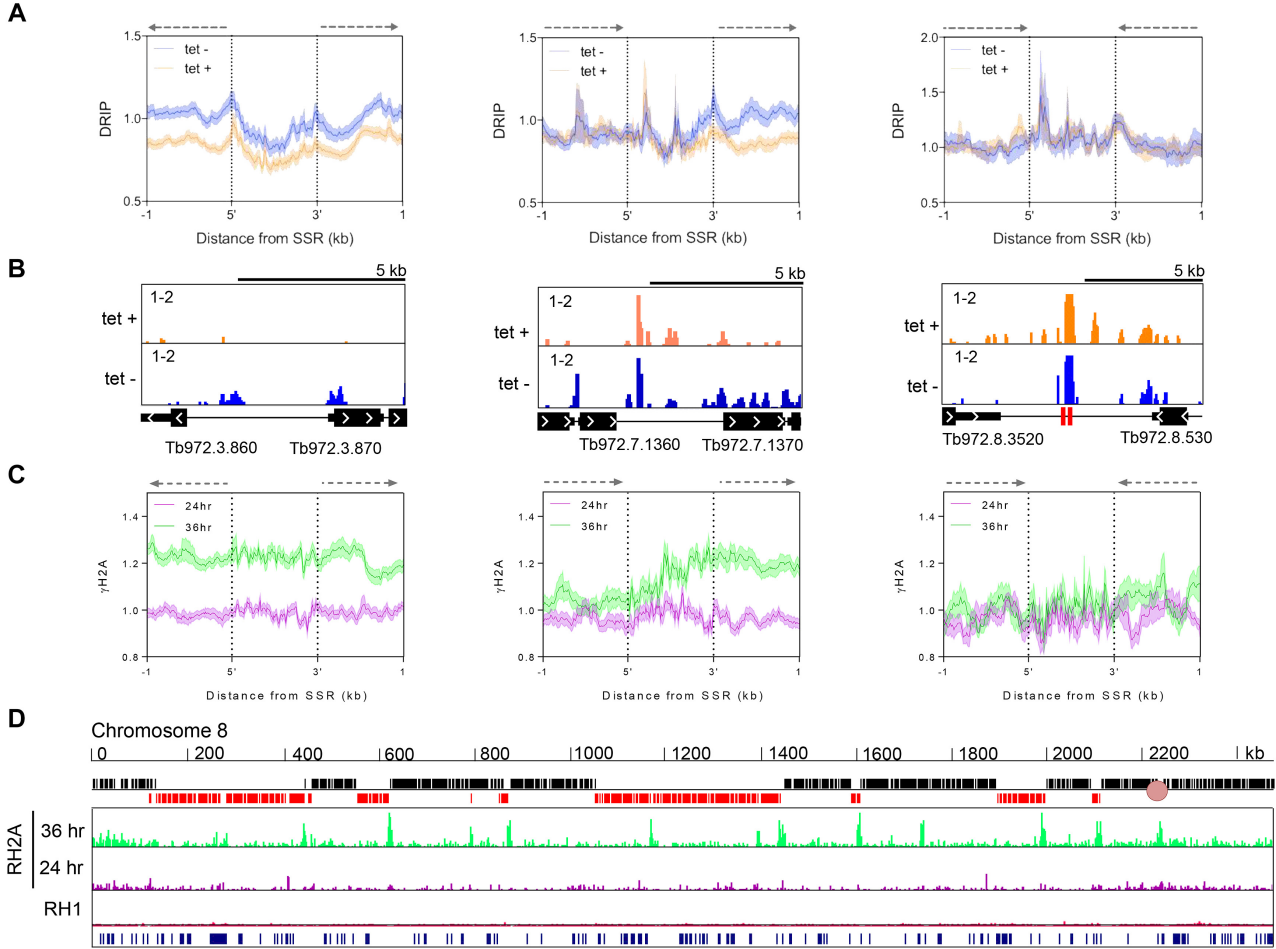
Altered RNA–DNA hybrid distribution and increased DNA damage at sites of RNA Pol II transcription initiation after TbRH2A depletion. (**A**) Average DRIP-seq signal is shown as metaplots plotted for TbRH2A RNAi uninduced (tet–, blue) and RNAi induced (tet+, orange) data sets over divergent (left), head-to-tail (middle) and convergent (right) SSRs (±1 kb). In all cases 5′ and 3′ denote SSR boundaries defined by flanking transcript coordinates. Transcription direction is shown above the plots by dashed arrows. Standard error is shown as shaded regions. (**B**) Example screenshots of DRIP-seq signal in tet+ and tet– cells at individual SSRs in each class; CDS (thick black), UTR (thin black lines) and snRNA/tRNA genes (red) are shown below the DRIP-seq tracks. (**C**) Metaplots of γH2A ChIP-seq signal in TbRH2A RNAi induced samples relative to uninduced is shown after 24 h (pink) and 36 h (green) of RNAi induction; average signal is plotted across SSRs as for (A). (**D**) γH2A ChIP-seq signal in induced relative to uninduced cells is also shown plotted across chromosome 8 after 24 h (pink) and 36 h (green) of growth; γH2A ChIP-seq in RH1 null mutant cells relative to wild-type cells is shown below (red) for comparison (scale 1–3 fold-change). Upper track shows genes on sense (black) and antisense (red) strands, and arrows highlight transcription direction; the lowest track shows tandem repeat sequences.

Previously, we described pronounced accumulation of DRIP-seq signal around the sites of transcription initiation in the SSRs that separate adjacent RNA Pol II PTUs (47). Here, mapping of DRIP-seq in the same loci revealed alterations in signal after tet-induced RNAi. Figure 3A shows DRIP-seq signal plotted over every SSR, with the loci separated into the following classes: divergent SSRs, representing sites of transcription initiation in both sense and antisense directions; convergent SSRs, which are sites of transcription termination; and head-to-to tail SSRs, where transcription both terminates and initiates on the same strand. Figure 3B provides detailed mapping at examples of each class of SSR. In all cases DRIP-seq signal in the uninduced cells showed a pronounced accumulation at the boundaries of the SSRs, corresponding to locations of transcription initiation, as well as some accumulation within the SSRs around where transcription might terminate, and at tRNA genes, as we described previously for WT cells (69). Upon depletion of TbRH2A, DRIP-seq signal no longer displayed such clear accumulation at the two regions of transcription initiation in divergent SSRs, or at the single site of transcription initiation in head-to-tail SSRs (right and central panels, Figure 3A, B). In contrast, DRIP-seq signal distribution appeared very comparable in induced and uninduced cells at convergent SSRs, or at the locations of transcription termination in head-to-tail SSRs (central and left panels, Figure 3A, B). To examine this effect of TbRH2A loss further, genes were separated into those predicted to be first within a PTU (i.e. proximal to the transcription start sites; *n*, 110) and all others (n, 8278; internal to the PTU) and the pattern of DRIP-seq examined around the genes’ ATG (Supplementary Figure S9). For all PTU-internal genes DRIP-seq abundance peaked upstream of the ATG and was depleted downstream of the ATG, with very similar profiles seen in the RNAi induced and uninduced cells. Moreover, the same R-loop accumulation was seen in uninduced cells upstream of the ATG at the first genes in the PTUs. In contrast, and consistent with the SSR mapping (Figure 3A, B), after TbRH2A RNAi DRIP-seq signal accumulation upstream of the ATG of transcription start site-proximal genes was notably less pronounced than was seen around the PTU-internal genes (Supplementary Figure S9A). In addition, this effect of TbRH2A RNAi on R-loop distribution at the first gene of the PTUs was not seen in TbRH1 null mutants (Supplementary Figure S9B). Thus, these data are consistent with loss of TbRH2A, and not TbRH1, mainly affecting R-loop abundance around sites of transcription initiation. Next, we re-grouped the SSRs according to whether or not they have been identified as origins of DNA replication (45) and re-analysed DRIP-seq distribution (Supplementary Figure S10). This analysis showed the same patterns of DRIP-seq signal across both types of SSRs in the RNAi induced and uninduced samples, indicating that it is transcription, and not DNA replication, of the SSRs that dictates the changed DRIP-seq signal distribution after loss of TbRH2A. Notably, the change in DRIP-seq pattern after RNAi against TbRH2A (Supplementary Figure S10A) was the opposite of the change in pattern seen in TbRH1 mutants compared with WT (Supplementary Figure S10B).

### TbRH2A RNAi induces DNA damage at transcription start sites in the core genome and results in gene expression changes

Given the highly localized changes in R-loop distribution after TbRH2A RNAi and the pronounced accumulation of γH2A, we next sought to ask if the effects are connected. To address this, we performed chromatin-immunoprecipitation (ChIP)-seq with anti-γH2A antiserum to map sites of accumulation of the modified histone, examining read depth distribution in tet-induced and uninduced cells grown for 24 and 36 h (Figure 3C, D; Supplementary Figure S11). In all cases, read depth in the IP samples was first normalized with pre-IP (input) samples, before fold-change in the RNA-induced cells was calculated relative to uninduced at each time point; Supplementary Figure S11 shows the resulting ratios of γH2A ChIP signal across the whole genome. Little change in γH2A localisation pattern was detected 24 hrs after TbRH2A RNAi induction (Figure 3C, D; Supplementary Figure S11), despite increased γH2A signal in IF and western analyses (Figure 2). However, after 36 hr of RNAi induction, clear accumulation in γH2A signal could be discerned at the boundaries of the PTUs in the core genome, an effect not seen in TbRH1 mutants (Figure 3D). To ask if this accumulation was specific for transcription start sites, the γH2A ChIP-seq data was mapped to SSRs grouped, as before, into divergent, convergent and head-to-tail classes (Figure 3C). Accumulation of γH2A was clearly apparent 36 h after RNAi across the divergent SSRs and around the sites of transcription initiation in the head-to-tail SSRs, but no such accumulation was detected at convergent SSRs. Hence, deposition of the DNA damage marker closely correlates with localized changes in RNA–DNA hybrid accumulation around transcription start sites, but with no such effects seen at termination sites. Thus, these data indicate that depletion of TbRH2A has localized effects, not seen after ablation of TbRH1 (47), which connect R-loops and DNA damage at sites of multigenic transcription initiation in *T brucei*.

**Figure 4.**
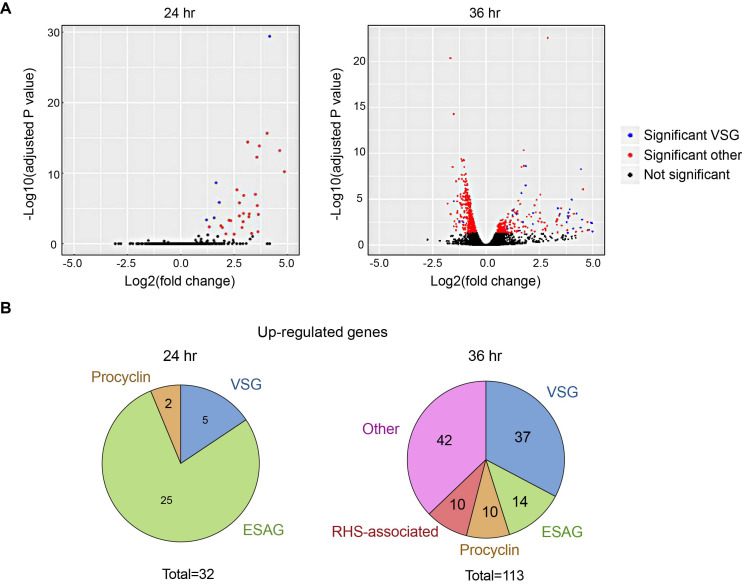
Gene expression is altered upon depletion of TbRH2A. (**A**) Volcano plots displaying differential expression of genes after TbRH2A knockdown. Each data point represents a gene. Genes were deemed significantly differentially expressed when RNA-seq indicated an adjusted *P* value <0.05 of gene-specific RNA in induced cells relative to uninduced. X-axes show the log2 fold-change between un-induced and induced after 24 h (left) and 36 h (right) of culture, and y-axes shows log_2_ adjusted p value. Data was generated with two independent replicates for each condition and time point. Significantly differentially expressed genes are shown in red or blue (denoting VSG); all other genes, including VSGs, are shown in black. (**B**) Number of genes significantly up-regulated in tet-induced TbRH2A RNAi cells relative to uninduced, after 24 (left) and 36 h (right) of growth, are shown annotated as VSGs, ESAGs, procyclin, RHS-associated, and other genes; total numbers are shown below each chart.

To ask if TbRH2A loss after RNAi affects gene expression, RNA-seq analysis was conducted, comparing mRNA abundance after 24 and 36 h of RNAi relative to uninduced control cells. After 24 h of TbRH2A RNAi, remarkably few changes in gene expression were seen: no genes displayed significantly reduced RNA abundance, while 32 showed significantly increased abundance (Figure 4A, B). More marked changes were found 36 h post-induction: 113 gene-specific RNAs significantly increased in abundance, and 396 were significantly reduced (Figure 4A, B). Interestingly, and as described further below, in keeping with our previous finding that R-loops can induce VSG switching in *T. brucei* (52), 30 of the 32 up-regulated genes after 24 h of TbRH2A depletion were annotated as VSGs (5) or ESAGs (25), a number that increased further after 36 h (37 VSGs, 14 ESAGs). At the same time more procyclin genes were significantly increased, suggesting effects on RNA Pol I transcription (Figure 4B), as well as increased numbers of RHS-associated genes, where R-loops could be mapped (Figure 1).

The remaining 42 up-regulated genes detected after 36 h RNAi, as well as the larger number of down-regulated genes, were more diverse in function (Supplementary Figure S12). However, GO term analysis revealed that several of the down-regulated genes were involved in small molecule biosynthesis pathways, and prominently represented were genes involved in nucleotide and ribonucleotide synthesis and salvage (Supplementary Figure S12). Other terms found to be over-represented in the down-regulated gene set were metabolic processing of other small molecules, including cellular carbohydrates, ketones and organic acids (Supplementary Figure S12). We could find no correlation between altered RNA abundance and gene position within a PTU.

### R-loops and DNA damage accumulate in VSG expression sites after RNase H2A depletion

**Figure 5.**
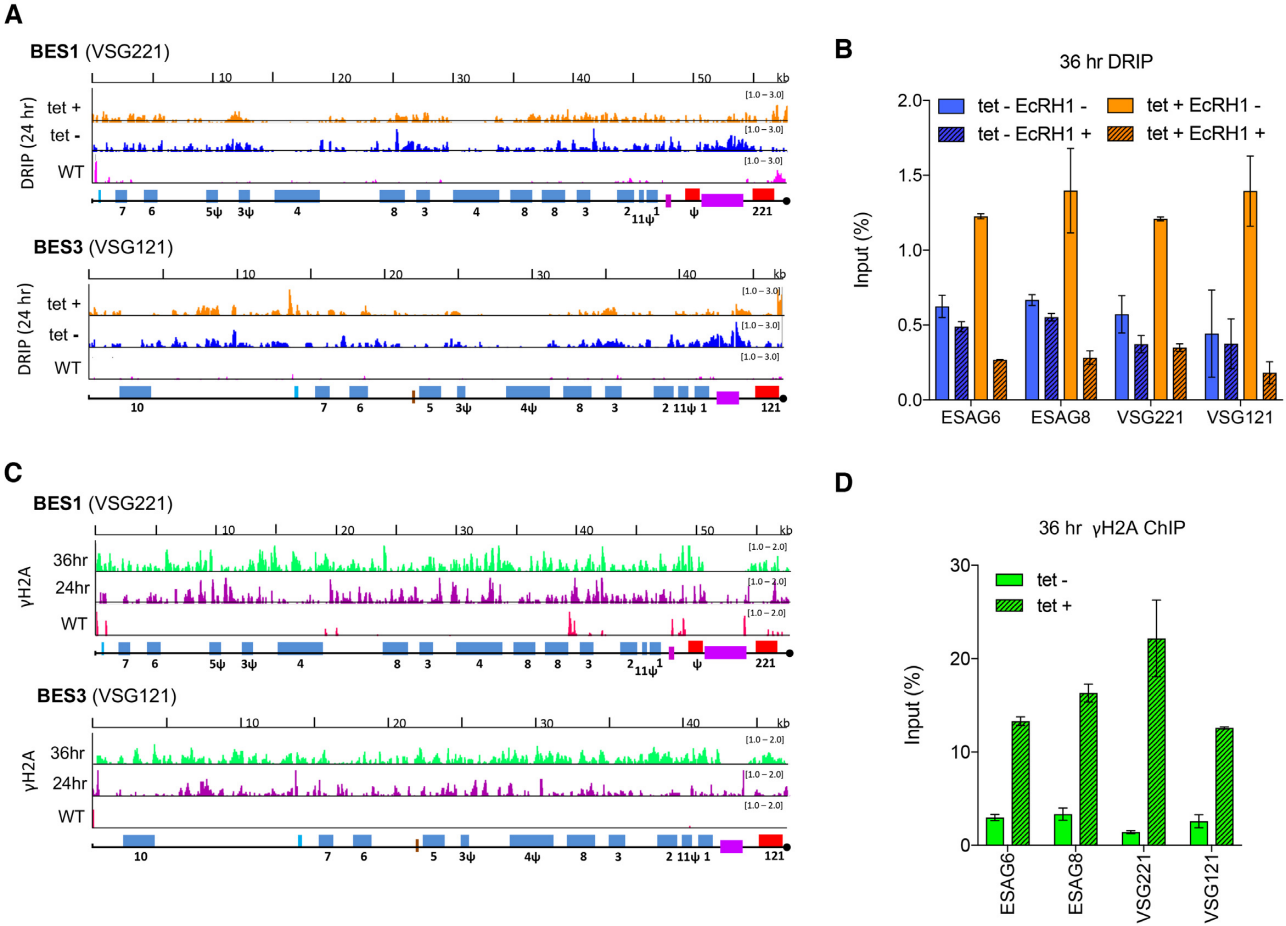
R-loop and yH2A levels increase across VSG BESs in cells depleted of TbRH2A. (**A**) Localisation of R-loops is shown in BES1 (active site of VSG transcription in WT cells) and BES3 (one normally transcriptionally silent site), comparing DRIP-seq signal in TbRH2A RNAi cells grown for 24 hr in the absence (tet–; blue) or presence (tet+; orange) of RNAi induction, and compared with WT cells (pink). BES features are shown as follows: promoter (aqua), ESAGs (blue, numbered), 70-bp repeats (purple) and VSGs (red); pseudogenes are indicated (ψ), and the end of the available BES sequence is denoted by a black circle. (**B**) DRIP-qPCR using primers targeting the sequences of ESAG6, ESAG8, VSG221 (BES1) and VSG121 (BES3), with or without *E. coli* RNase HI (EcRHI) treatment, showing the percentage of amplification in the IP sample relative to input from tet induced (tet+) and uninduced (tet–) cells grown for 36 h. Error bars display SEM for three replicates. (**C**) yH2A ChIP-seq signal enrichment is shown mapped to BES1 and BES3 as a ratio of reads in tet-induced samples relative to uninduced (each first normalized to the cognate input sample) after 24 (purple) and 36 (green) h growth; γH2A ChIP-seq signal (normalised to input) is shown in WT cells for comparison (pink). (**D**) γH2A ChIP-qPCR, as in (B): data is shown for tet induced (tet+) and uninduced (tet–) cells after 36 hr of growth. Error bars display SD for two replicates.

We have previously reported increased BES-associated R-loops in *TbRH1* null mutant BSF *T. brucei*, coinciding with increased levels of VSG switching and increased replication-associated DNA damage (52). To ask if these antigenic variation-directed effects are limited to TbRH1 activities, we first plotted DRIP-seq before and after TbRH2A RNAi across all the available BES sequence (48), comparing the mapping to DRIP-seq in WT cells (Figure 5A, B; Supplementary Figure S13). To limit cross-mapping of short reads to related BES we applied MapQ filtering (59). After 24 h, both uninduced and induced cells showed substantially increased DRIP-seq signal across both the active (BES1) and all inactive BESs (Figure 5A, B; Supplementary Figure S13) in comparison with WT. Increased signal in the uninduced cells is presumably due to leaky expression of the TbRH2A-targetting stem-loop RNA, consistent with the slight reduction in TbRH2A RNA (Figure 1A) and small changes in VSG expression (see below). Why the DRIP-seq mapping did not reveal further increases in levels of R-loops in the BES after RNAi is unclear, but may reflect confounding issues of read mapping after data normalization (70), since DRIP-qPCR suggested increased R-loop levels in RNAi induced cells relative to uninduced (Figure 5B). Strikingly, the pattern of R-loop distribution in the BES compared well with TbRH1 null mutants, with prominent signal in the 70 bp-repeats and little localisation to sequences between the ESAGs (Figure 5A, Supplementary Figure S13), distinguishing the RNA–DNA hybrid distribution from the RNA Pol II PTUs (Supplementary Figures S7 and S8). To establish if the DRIP-seq detects RNA–DNA hybrids, DRIP-qPCR was performed with samples prepared after 36 h of growth with and without tet induction, including, in each case, a parallel IP that was treated with *E. coli* RNase HI (EcRH1) to degrade the RNA within the hybrids (Figure 5B). ESAGs 6 and 8 were targeted to examine R-loops within all BES, while primers recognizing VSG221 and VSG121 were used to examine the predominantly transcriptionally active (BES1) BES and one transcriptionally silent BES (BES3), respectively. In all cases, increased RNA–DNA hybrids were detected in the RNAi induced samples relative to uninduced and treatment with EcRH1 reduced IP levels for nearly all samples, validating detection of R-loops (Figure 5B).

To ask if the BES R-loops also correlate with damage in this component of the genome, we next mapped γH2A ChIP-seq to the BESs, comparing signal fold-change in the RNAi-induced samples relative to uninduced (Figure 5C, D; Supplementary Figure S14). These data revealed a number of features. First, extensive accumulation of γH2A signal was seen across the entire length of both active and inactive BESs (Figure 5C), as confirmed by ChIP-qPCR (Figure 5D). The extent of the accumulation, and the presence of phosphorylated H2A in both active and inactive BES, is distinct from the γH2A ChIP-seq profile seen in *TbRH1* null mutant cells (52), where γH2A is only significantly mapped to telomere-proximal regions of the active BES, not the silent sites. Second, accumulation of γH2A in the BES was clearly discernible 24 h post RNAi induction, unlike in the core genome, where signal was only seen after 36 h RNAi. In addition, accumulation of the modified histone was not limited to, or more pronounced at, the promoter of the BES. Thus, loss of TbRH2A has a more rapid and widespread effect on BES integrity than is seen in the core genome. Third, in contrast with the strong DRIP-seq signal across the 70 bp repeats after TbRH2A loss, γH2A ChIP-seq was notably limited on this BES feature relative to all other parts of the BESs, perhaps indicating a particular effect of repeat composition on generation or spreading of the modified histone.

Finally, to ask if the above effects are limited to the BES, we mapped the DRIP-seq and γH2A ChIP-seq data to the rRNA genes, which are also transcribed by RNA Pol I (Supplementary Figure S15). We saw the same patterns of R-loop distribution before and after 24 h of RNAi as seen in the BES, and the same accumulation of modified histone after RNAi. Thus, coincident accumulation of R-loops and DNA damage appears to be a common effect at sites of RNA Pol I transcription, which differs from the localized damage accumulation seen after TbRH2A loss at RNA Pol II transcription start sites.

### Loss of RNase H2A leads to changes in VSG expression

**Figure 6.**
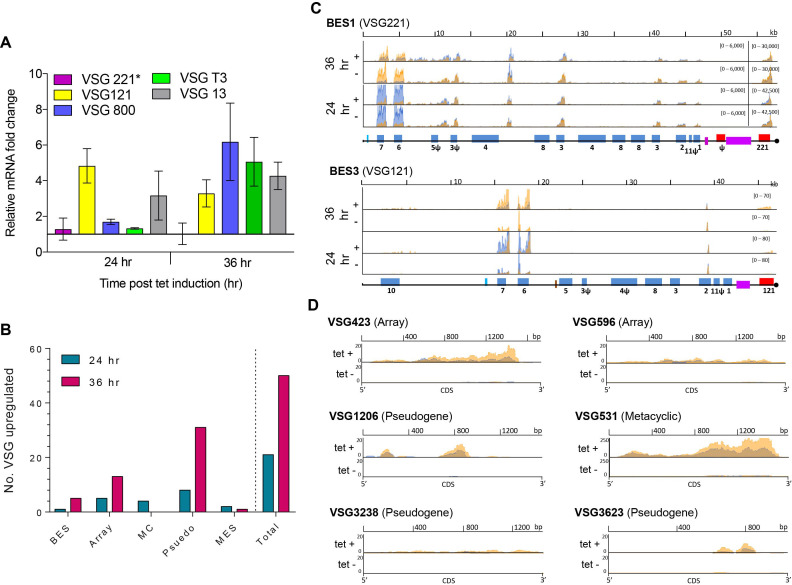
Depletion of TbRH2A results in increased transcription of silent VSGs. (**A**) Graph of RNA levels for five VSGs, determined by RT-qPCR, in tet-induced TbRH2A RNAi cells, plotted as fold-change relative to levels of the cognate VSG RNA in uninduced cells after both 24 and 36 h of culture; VSG221 (pink) is in the active BES (BES1) of WT cells, while VSG121 (yellow), VSG800 (blue), VSGT3 (green) and VSG13 (grey) are in silent BESs; error bars show SD for three independent experiments. (**B**) Graph depicting the number of VSG genes whose RNA levels display significant upregulation in RNAi-induced RNA-seq samples relative to uninduced, both 24 and 36 h after growth; the total number is sub-categorised depending on whether the VSGs have been localized to the BES, are intact genes in the subtelomeric arrays (array), are in minichromosomes (MC), are pseudogenes (pseudo), or are in a metacyclic VSG ES (MES). (**C**) Normalized RNA-seq read depth abundance (y-axes) is plotted for two independent replicates (overlaid orange and blue) in TbRH2A RNAi parasites after 24 and 36 hr of growth, with (tet+) or without (tet–) RNAi induction. Read depth is shown relative to gene position (x-axes) for BES1 and BES3. (**D**) As in (C), showing RNA-seq read depth abundance (y-axes) across a selection of non-BES VSG CDS (x-axes), after 36 h of growth.

As discussed above, RNA-seq analysis revealed increased RNA levels of several VSG, ESAGs and procyclin genes in response to TbRH2A depletion (Figure 4). To examine this in more detail, we first used RT-qPCR to examine levels of VSG221, which is expressed from the predominantly active BES, and four VSGs housed in normally transcriptionally silent BESs, after 24 and 36 h of TbRH2A RNAi (Figure 6A). Levels of VSG221 transcript did not change significantly after TbRH2A knockdown, but levels of two silent VSGs increased after 24 h RNAi, and all four silent VSG levels increased relative to uninduced samples after 36 h of TbRH2A knockdown (Figure 6A). To investigate changes in VSG transcription more widely, RNA-seq reads were mapped to all the BESs (Figure 6C, Supplementary Figure S16), as well as a collection of 2470 VSG sequences described in the *T. brucei* Lister 427 genome (60) (Figure 6B, D), and differential expression analysis was repeated. 20 VSG sequences were found to be significantly up-regulated after 24 hr of TbRH2A depletion, a cohort that increased to 50 VSGs after 36 h of RNAi (Figure 6B). Of these, 40% and 60% were classified as pseudogenes in the 24 and 36 h samples, respectively. Of the remaining VSG sequences up-regulated after 24 h of RNAi, 25% were classified as intact genes found within subtelomeric arrays, 20% as intact genes associated with mini-chromosomes, 10% as metacyclic (M) ES VSGs, and the remaining 1 VSG as BES-housed (Figure 6B). After 36 h, a similar proportion of up-regulated VSGs were array-associated (26%), 10% were housed in the BESs, and 2% (1 VSG) were MES-associated (Figure 6B); no mini-chromosome associated VSGs were significantly up-regulated at this time point. Hence, VSG sequences from across the diverse genome repertoire were found to be transcribed after TbRH2A depletion. Within the silent BESs, RNA-seq mapping suggested that not only did VSG RNA levels increase after RNAi, but also levels of promoter-proximal ESAGs (most clearly seen as ESAG 6 and 7; Figure 6C), explaining the increased levels ESAG-associated reads described in Figure 4.

**Figure 7.**
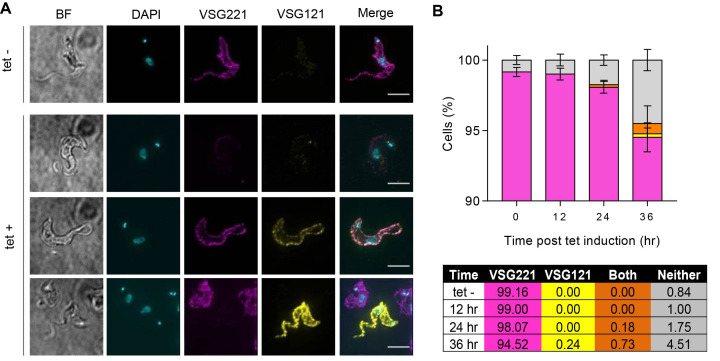
Depletion of TbRH2A induces switching of the VSG coat. (**A**) Co-immunofluorescence imaging of VSG221 (pink) and VSG121 (yellow) surface expression. Example images are shown of cells after 24 h of culture without (tet–) or with (tet+) RNAi induction (Scale bar, 5 μm). (**B**) Graph of the percentage of uninduced (tet–, all time points) and RNAi-induced cells (after 12, 24 and 36 h of culture with tet) expressing only VSG221 (pink) or only VSG121 (yellow) on their surface, as well as cell with both (orange) or neither (grey) of the two VSGs on their surface, as determined by co-immunofluorescence imaging with anti-VSG221 and VSG121 antiserum. >200 cells were analysed for each time point and three experimental replicates (error bars denote SEM). The table below shows the average percentage of the three replicates in each case.

In order to ask if changes in VSG RNA levels in response to TbRH2A depletion extended to VSG protein changes on the parasite surface, expression of two VSGs, VSG221 (active BES1) and VSG121 (inactive BES3), was analysed via immunofluorescence with specific antisera (Figure 7A, B). Unpermeabilised cells were probed for expression of both VSGs after 12, 24 and 36 h growth with and without tet-induction of RNAi. In the absence of RNAi, across all time points, 99% of cells expressed just VSG221 (∼99%) (Figure 7B), and 1% did not stain for either VSG. The small loss of VSG221 expressers in the absence of tet-induction is probably due to leaky RNAi, and consistent with the other phenotypes described above, since 100% of parental (WT) *T. brucei* cells exclusively express VSG221 (52). After 12 h of induction, singular VSG expression did not significantly change. However, after 24 h of RNAi nearly 2% of cells did not stain for either VSG, and ∼0.2% expressed both VSGs simultaneously. By 36 h of induction, the number of cells expressing both VSG221 and VSG121 increased further to ∼0.7% of the population, parasites expressing neither VSG also increased to ∼4.5% and, at this time point alone, a small number of cells (∼0.2%) could be detected that expressed VSG121 but not VSG221. Taken together, these data indicate that loss of TbRH2A results in an increased frequency at which *T. brucei* cells inactivate expression of VSG221, as well as causing loss of the controls that ensure monoallelic expression of a single VSG in one cell.

## DISCUSSION

RNase H enzymes that hydrolyse RNA within an RNA–DNA hybrid or remove ribonucleotides in DNA are ubiquitous in nature, with all organisms appearing to encode at least one RNase H, and most encoding two types of RNase H (26,32). Though such ubiquity suggests crucial roles, including in DNA replication, repair and transcription, loss of RNase H has so far only been described as being lethal during development in mammals (71) and *Drosophila melanogaster* (72). In this study, we describe the roles played by RNase H2 in *T. brucei*, revealing the first example, to our knowledge, of an RNase H being essential in a single-celled eukaryote. The lethality we describe after TbRH2A loss contrasts with the non-essentiality of *T. brucei* RNase H1 (47,52) and our data indicate that at least one explanation for this separation of function is that TbRH2 plays a specific and potentially novel role in processing R-loops at sites of multigenic RNA Pol II transcription initiation. Despite this distinction between the two *T. brucei* RNase H enzymes, we also reveal overlapping roles in targeting R-loops in RNA Pol I transcription units, contributing to the control and execution of immune evasion by antigenic variation (52). Finally, gene expression analysis reveals changes in expression of genes involved in nucleotide and ribonucleotide synthesis and salvage pathways after TbRH2 loss, perhaps indicating that impairment of the parasite RNase H2 has cellular effects that may have some parallels with phenotypes described in humans with the autoimmune syndrome AGS (73), which can be caused by RNase H2 mutation.

RNAi-mediated depletion of TbRH2A led, within 24 h (∼3–4 cell cycles), to a growth arrest of BSF *T. brucei* cells that was accompanied by a pronounced impairment in the cell cycle, manifest initially as accumulation of 1N2K (G2/M phase) cells and then by the appearance of aberrant cells that failed to effectively divide their nucleus. Over the same period, TbRH2A RNAi resulted in increased expression of γH2A, which could be detected throughout the nucleus of most cells in the population, indicating more severe levels of nuclear genome damage than are seen after ablation of TbRH1 (52). Thus, both *T. brucei* RNase H enzymes examined to date appear to act in the nucleus (52,69) and we have found no evidence that either provides mitochondrial functions seen in other eukaryotes (74). Unlike in yeast, where both RNase H genes can be deleted (33,34), lack of continued proliferation of *T. brucei* cells after depletion of TbRH2A appears more comparable with truncated mammal embryogenesis seen in RNase H2B and RNase H2C mutants (71,75). Indeed, the nucleus-focused phenotypes after loss of RNase H2A in *T. brucei* are reminiscent of increased levels of γH2AX in cells from RNase H2 mutant mice (71,75), and with fibroblasts taken from RNase H2B mutant mice showing slowed growth and accumulation in the G2/M phase of the cell cycle (75). However, DNA content analysis revealed some differences when comparing RNase H2 mutant mouse cells and TbRH2A-depleted *T. brucei* parasites: flow cytometry and EdU incorporation analysis indicate that loss of TbRH2A does not prevent DNA synthesis, whereas RNase H2 mutant mouse embryonic cells display reduced incorporation of EdU (71). While this difference may only reflect variation between mouse and *T. brucei* cells in eliciting a cell cycle checkpoint in response to damage resulting from loss of RNase H2, perhaps due to changes in DNA damage signalling, it is also possible that genome replication is not the process primarily affected by TbRH2A loss in *T. brucei*. A less likely explanation, given the increased DNA content seen during flow cytometry in TbRH2A RNAi cells, is that *T. brucei*, unlike mice, enacts robust DNA repair synthesis, such as break-induced replication, in response to lesions caused by loss of RNase H2.

Unlike RNase H1, RNase H2 has the capacity to excise ribonucleotides incorporated into the genome by initiating ribonucleotide excision repair (30,32,76), and lack of this activity has been proposed to underlie the embryonic lethality of mice RNase H2 mutants (71,75). However, yeast RNase H2 mutants also display increased levels of DNA-embedded ribonucleotides, leading to increased genome instability (33,34), but survive. Equally, *D. melanogaster* RNase H1 mutants, which are likely to be unaltered in their capacity to remove ribonucleotides from the genome, display curtailed development during metamorphosis as a result of altered gene expression (72). In this context, we suggest that the highly localised accumulation of γH2A and altered R-loop abundance we describe at sites of multigenic transcription initiation in the genome core after TbRH2A RNAi may provide an explanation for the importance of RNase H2 in *T. brucei*. We have shown previously that, amongst the widespread distribution of R-loops across the *T. brucei* genome, RNA–DNA hybrids display a clear association with the ∼200 mapped sites of RNA Pol II transcription initiation (47), with a strong correlation in localisation relative to epigenetic features found at such SSRs (42,44,77). We now show that TbRH2A depletion results in pronounced γH2A accumulation only at these RNA pol II transcription initiation sites, not at termination sites or within the PTUs, and that these same loci are the only RNA Pol II-transcribed regions in the genome where DRIP-seq detects pronounced changes in R-loop distribution after RNAi. Strikingly, neither accumulation of damage nor changes in R-loop patterns is seen at the these sites in TbRH1 null mutants (47). The combined alteration of R-loop pattern and accumulation of damage at these loci argue that these phenotypes are not the result of localized, increased levels of embedded ribonucleotides, but instead that RNase H2 has a specific role, not possessed by RNase H1, in processing R-loops associated with RNA Pol II transcription initiation.

Why altered R-loop processing after loss of TbRH2A might lead to damage at transcription start sites is unclear, at least in part because we do not know the nature of the DNA lesion(s) that leads to γH2A accumulation. In addition, though there is clear evidence that R-loops are associated with genome instability, the processes that lead from an R-loop to DNA damage are less clearly understood (10,78,79). Nonetheless, a number of explanations might be considered. One simple explanation is that loss of RNase H2 activity increases R-loop stability at transcription start sites, causing the extruded DNA strand to be more susceptible to breakage. However, this seems unlikely, since we appear to see loss of R-loops at the same loci after TbRH2A RNAi and previous work has shown that single-strand breaks can increase R-loop formation (80). An alternative explanation may be that RNase H2 provides a specific route for clearance of R-loops at the sites of transcription initiation and in its absence other factors attempt to remove the RNA–DNA hybrids incorrectly, leading to increased damage and reduced R-loop levels (81). An RNase H2-specific activity of this sort would explain why loss of TbRH1 does not cause such phenotypes, but the nature of such an activity and how it is enacted only by RNase H2 and not RNase H1 is unclear. At this stage, we cannot rule out that such an activity is due to novel interaction of TbRH2A, or the predicted *T. brucei* RNase H2 trimer, with unknown factors. Indeed, we also cannot rule out that loss of *T. brucei* RNase H2 activity allows RNase H1 to act in its place, resulting in DNA damage. A final explanation is that failure to efficiently process the R-loops due to loss of TbRH2A leads to mutagenic effects due to impairment of other processes. For instance, inefficient or inaccurate removal of R-loops might impede RNA Pol II’s movement away from the transcription start site, leading to a blockage of DNA replication (25) or activation of nucleotide excision repair due to stalling of the RNA Pol (82), both of which might lead to localized DNA lesions marked by γH2A. In this regard, recent work by Costantino and Koshland (83) may be relevant. Like the data present here, these authors mapped DNA damage in yeast cells impaired in R-loop removal, providing clear evidence for genome-wide correlation between sites of RNA–DNA hybrid formation and localisation of a damage marker (in their case, Rad52). Furthermore, not all sites of R-loop formation in yeast led to damage, consistent with the localised accumulation of γH2A at only a fraction of *T. brucei* genome sites that display R-loop enrichment (69). However, despite these similarities, significant differences are found in our data compared with the study by Costantino and Koshland (83). First, despite the fact that both studies detected damage accumulation at rRNA sites of R-loop accumulation, such an effect in yeast requires mutation not only of both RNase Hs but also Senataxin or Topoisomerase I, whereas in *T. brucei* loss of only RNase H2 function leads to damage. Second, there was no evidence that the R-loop-associated damage in yeast shows the precise co-localisation with sites of RNA Pol II transcription initiation that we have mapped in *T. brucei*. Thus, though it is conceivable that the damage we detect via γH2A might also be sites of single-stranded DNA bound by Rad52 (83), distinct routes of damage formation are likely, and our experiments implicate R-loops and RNase H2 in hitherto unexplored aspects of RNA Pol II transcription. Intriguingly, mounting evidence has linked the generation of DNA breaks and the action of DNA repair factors in transcriptional activation (84–86), including regulating elongation of RNA Pol at mammalian protein-coding and non-coding RNA genes (87,88), and altering chromatin to activate gene expression during *Caenorhabditis elegans* embryogenesis (89). Thus, our data suggest it is possible that DNA breaks are also a feature of transcription initiation in *T. brucei*, with their extent or persistence increased by loss of RNase H2. Mapping RNA Pol II by ChIP has revealed accumulation at transcription start sites, consistent with pausing (44), which correlates with R-loops at these sites (47). It is important to note, however, that there is no evidence for *T. brucei* RNA Pol II transcription being controlled at the point of initiation (44), so whether the precise association we observe between RNase H2-associated DNA damage and R-loop levels at the start of multigenic transcription in *T. brucei* might have parallels with single gene, regulated transcription in other eukaryotes is currently unknown. Nonetheless, R-loops are readily detected at CpG island promoters in the human and mouse genomes (90,91). Moreover, near genome-wide multigenic RNA Pol II transcription is common to all kinetoplastids (39), meaning it is likely that RNase H2 plays related roles in transcription initiation throughout this grouping of microbes and may be fertile ground for new therapies against diseases caused by the parasites.

The RNA-seq we describe here evaluates differential expression of gene-specific transcripts before and after TbRH2A RNAi and revealed two aspects of TbRH2 function, neither of which explain the localized effects of RNase H2 loss at transcription start sites. First, as discussed more fully below, the genes most rapidly and most strongly affected by loss of TbRH2 are transcribed by RNA Pol I, not RNA Pol II. Second, and perhaps surprisingly, only a relatively small cohort of differentially expressed RNA Pol II transcripts were found, many associated with metabolic activities, including prominent changes in genes implicated in nucleotide metabolism. Moreover, we found no evidence that such genes were found at specific parts of the multigenic transcription units, or that genes with the most pronounced reduction in RNA levels had notably high or low levels of R-loops either before or after TbRH2A RNAi (data not shown). Thus, the RNA-seq data do not provide evidence that loss of TbRH2A results in global or localized changes in RNA levels, but it may be necessary to examine nascent RNA levels to examine this further, or to include exogenous RNA to normalize the RNA-seq data between induced and uninduced parasites. Nonetheless, the differential changes revealed by RNA-seq in *T. brucei* may be worth exploring further. AGS disease in humans is caused by mutation of RNase H2, as well as several other genes that encode enzymes with nucleic acid or nucleotide targeting activities (35). Mouse models of AGS carrying RNase H2 mutations have shown that activation of the cyclic GMP-AMP synthase (cGAS)-STING immune sensing pathway elicits a type I interferon response associated with AGS pathological symptoms (36,37). Since the cGAS-STING pathway normally detects cytosolic DNA, mediating one arm of the innate immune response against pathogens (92), it is it is considered likely that increased abundance of DNA with embedded ribonucleotides or RNA–DNA hybrids aberrantly activate the cGAS-STING pathway during AGS (36,37). It is conceivable that the putative downregulation of nucleotide and ribonucleotide synthesis and salvage pathways after TbRH2A RNAi implicates RNA Pol II transcription start site-associated R-loops with nucleotide metabolic regulation, or that such a change reveals a role for *T. brucei* RNase H2 in RER, a pathway not so far experimentally examined in the parasite.

Despite the pronounced, localised effect of TbRH2A RNAi at sites of RNA Pol II transcription initiation, RNA-seq revealed that a stronger and earlier effect was exerted on protein-coding genes expressed by RNA Pol I. Most notably, TbRH2A loss resulted in increased RNA levels of previously silent VSGs, leading to changes in the surface coat. As these changes in VSG expression were concomitant with accumulation of R-loops and DNA damage in the telomeric VSG BES, and reflect similar findings described in *TbRH1* null mutants (52), the data provide further evidence that R-loops acted upon by RNase H are an important driver of antigenic variation through VSG switching. Similarities and differences in the VSG-associated phenotypes seen after TbRH2A RNAi and ablation of TbRH1 suggest overlap in the function of the two enzymes in this reaction, but also some divergence. In both TbRH2A-depleted and *TbRH1* null cells, R-loop levels appeared to increase to similar levels in both the active and silent BES. Though these data suggest that loss of either RNase H impairs resolution of BES R-loops and leads to VSG switching, how the RNA–DNA hybrids accumulate in the silent ES, which are not transcribed to the same extent as the main active ES, remains unclear. Despite this similarity, levels of BES damage measured by γH2A ChIP differed: whereas loss of TbRH1 results mainly in increased damage towards the telomere of the active BES, TbRH2A depletion caused greater amount of yH2A accumulation throughout both the active and silent BES. This difference may reflect a greater impact of TbRH2A depletion on transcription, although greater damage induction due to persistence of ribonucleotides in the BES cannot be ruled out; indeed, increased incorporation of uracil into DNA, due to loss of uracil-DNA glycosylase, has been shown to lead to DNA lesions and VSG switching (93). Nonetheless, two pieces of evidence suggest that loss of TbRH2A affects BES transcription more that ablation of TbRH1. First, whereas RNA-seq only revealed increased expression of VSGs in *TbRH1* mutants, increased levels of RNA from promoter-proximal ESAGs, as well as from BES VSGs, was detected by the RNA-seq described here. Second, in TbRH2A depleted parasites, significantly higher numbers of cells were found that expressed two VSGs simultaneously on their surface compared with the same IF analysis of *TbRH1* null mutant cells. Hence, loss of RNase H2 compromises more strongly the strict monoallelic expression of VSG BES normally employed by *T. brucei* (94). Whether this is because the TbRH2 complex recruits factors involved in monoallelic control, while TbRH1 does not, is worthy of investigation; for instance, it is known that RNase H2B in other eukaryotes interacts with PCNA (95). We have argued that DNA damage induced in the BES by R-loops, and accentuated by loss of TbRH1, is a plausible route for the initiation of recombination of silent VSGs (52). This argument is supported here by the finding that TbRH2A RNAi also led to increased BES damage and expression of silent VSGs from throughout the repertoire, including intact subtelomeric genes, minichromosome genes and pseudogenes, which are unlikely to be transcribed without movement to the BES. Nonetheless, how, when and where BES R-loops are converted into DNA damage, and the nature of the lesions that arise, remain open questions.

## DATA AVAILABILITY

Sequences used in the mapping have been deposited in the European Nucleotide Archive (accession number PRJEB22057).

## Supplementary Material

gkz644_Supplemental_File
