# Supplementary material for: Trypanosoma brucei ribonuclease H2A is an essential R-loop processing enzyme whose loss causes DNA damage during transcription initiation and antigenic variation
Source: Nucleic Acids Res. 2019 Jul 27;47(17):9180–97. doi: 10.1093/nar/gkz644 (PMC6753483; doi:10.1093/nar/gkz644)
Supplement: gkz644_Supplemental_File [file gkz644_supplemental_file.pdf]

Supplementary figure legends

Table S1. Results of BLAST analysis using the predicted polypeptides of the three putative subunits of RNase H2 found in the genome of *Trypanosoma brucei*.

Figure S1. Protein sequence analysis of putative *T. brucei* type 2 RNase H proteins. A) Structural models of putative RNase H2 subunits A (Tb427.10.5070), B (Tb427.01.4220) and C (Tb427.01.4730), as predicted by InterPro analysis. Conserved domains are shown, and active site and catalytic residues are highlighted as white boxes and red diamonds above predicted domains, respectively. B) Amino acid alignments of putative RNase H2A catalytic proteins from *T. brucei*, *T. cruzi*, *L. major*, *E. coli* and *T. maritina*. Predicted sequences of the RNase H (H-)domain is highlighted in blue, active site residues in cyan, and catalytic residues are indicated by red dots.

Figure S2. TbRH2A is a nuclear protein in bloodstream form *T. brucei* parasites. A) Western blot detection, using anti-HA antiserum, of C-terminally 6HA-tagged TbRH2A protein expressed from its endogenous locus in clonal bloodstream form *T. brucei*. A sample from untagged wild-type (WT) parasites is shown for comparison along with the expected protein size (kDa). B) Representative immunofluorescent images of *T. brucei* parasites expressing TbRH2A-6HA; WT cells are shown for comparison. Anti-HA signal is shown in magenta and DAPI staining in cyan. The cell outline is shown by differential interference contrast microscopy (DIC). Scale bar, 5μm. C) Super-resolution structured illumination imaging of TbRH2A and nuclear DNA (magenta and cyan respectively in the merged, uncoloured images), detected with anti-HA antiserum and DAPI respectively. A respective cell of each cell cycle stage is shown along with graphs plotting length across the nucleus (x-axis, pixels) versus mean pixel intensity at each point (y, arbitrary units) for DAPI (cyan) and TbRH2A (magenta). Scale bars, 5μm. D) Graphs depicting mean fluorescence intensity (arbitrary units, a.u.) of DAPI (left, cyan) and anti-HA- staining (right, magenta) to detect TbRH2A expression. Cells are separated by discernible cell cycle stages (determined by number of nuclear (N) and kDNA (K) signals visualised by DAPI staining; 1N1K, 1N1elongatedK (1N1eK), 1N2K and 2N2K). Each data point denotes the mean fluorescence of an individual cell and the median values (horizontal lines) and interquartile range (error bars) are shown. Significance was determined by the Kruskal-Wallis non-parametric test: (**) p-value <0.01, (***) p-value <0.001, (****) p-value < 0.0001.

Figure S3. DNA synthesis occurs in the presence of DNA damage upon depletion of TbRH2A in *T. brucei*. A) Immunofluorescence detection of nucleic acid (DAPI staining; cyan), thymidine analogue EdU incorporation (via Click-IT chemisty; yellow) and γH2A (anti-γH2A antiserum; magenta) in TbRH2A RNAi parasites grown in either the absence (tet -) or presence of tet-induction (tet +) for 12, 24 or 36 hr. EdU and γH2A signal colocalization within several cells is also shown as merged images.

Scale bars, 5µm. B) SR-SIM images of DAPI, γH2A and EdU staining are shown, along with 3D reconstructions (model), of TbRH2A RNAi cells grown in the absence (tet -) or presence (tet +) of tet-induction. Scale bars, 1 µm.

Figure S4. DNA synthesis continues after depletion of TbRH2A in *T. brucei*. Graphs show fluorescence intensity of the nucleus after DAPI (left, blue) and EdU- staining (right, orange) at different time points after growth with (Tet+) or without (Tet-) induction of RNAi against TbRH2A. Each dot represents the mean pixel intensity (arbitrary units) of the nucleus of an individual cell; median values (horizontal lines) and interquartile range (error bars) are shown.

Figure S5. DRIP-seq signal across the 11 Mb-sized chromosomes of *T. brucei* before and after TbRH2A depletion. DRIP-seq signal for TbRH2A RNAi parasites grown without (blue) and with (orange) tet-induction for 24 hr is plotted as fold-change of IP sample coverage relative to input sample coverage (scale: 1-4 fold change). Upper track shows genes encoded in the sense (black) and antisense (red) directions. Known centromeres are shown as peach ovals. Lowest track shows predicted tandem repeat sequences.

Figure S6. Distribution of DRIP enriched regions across the 11 Mb-sized chromosomes. The distribution of DRIP-seq enriched regions (> 1.2 fold change DRIP signal relative to input signal) in TbRH2A RNAi tet-induced (tet +) and uninduced (tet -) parasites. The composition of the Mb-sized chromosomes is shown to the left for comparison.

Figure S7. Distribution of DRIP enriched regions across the RNA Pol II transcribed polycistronic units. A) The distribution of DRIP enriched regions between CDS, 5' UTR, 3' UTR and intergenic sequences within the RNA Pol II transcribed PTUs is shown for WT , TbRH2A RNAi uninduced (tet -) and induced (tet +) cells. Total numbers of DRIP enriched regions defined are displayed below plots. B) DRIP-seq signal coverage (normalised to input sample coverage; scale 1-3 fold-change) across an example regions of a PTU within Mb chromosome 2, for TbRH2A RNAi uninduced (tet -) and induced (tet +) samples. CDS regions are indicated by thick black lines below tracks, UTR sequences are indicated by thin black lines, and white arrows indicate transcription direction.

Figure S8. DRIP-seq signal is enriched in regions flanking CDS of RNA Pol II transcribed genes. Heatmaps of DRIP-seq signal (normalised to input signal) across each RNA Pol II transcribed protein-coding CDS, plus 1 kb of upstream and downstream flanking region, are shown for WT, TbRH2A RNAi uninduced (tet -) and induced (tet +) cells. Profiles of the average DRIP-seq of all CDS is shown above each heatmap.

Figure S9. DRIP-seq signal mapping across the translational start sites of RNA Pol II transcribed genes. A) Average DRIP-seq signal coverage (normalised to input coverage; x axes) is plotted across 1

kb surrounding the ATG start sites (y axes) of the first gene in each RNA Pol II transcribed PTU (left), and all other genes contained with the PTUs (right). Average signal is shown TbRH2A RNAi uninduced (blue; tet -) and induced (orange; tet +) cells. Shaded areas show SEM. B) As in A, but showing the same data for wild type cells (WT, purple) and *T. brucei* RNase H1 null mutants (green, *Tbrh1*-/-).

Figure S10. Levels of DRIP-seq signal are not altered in DNA replication origins SSRs compared with non-origin SSRs. A) The average DRIP-seq signal coverage (normalised to input sample coverage) is plot across SSRs, plus 1 kb of upstream and downstream flanking regions, which are known origins of replication (ORI) for TbRH2A uninduced (blue; tet -) and induced (orange, tet +) cells. The 5' and 3' boundaries of each SSR were defined as the end and start of flanking transcripts. Shaded areas represent SEM. An example of DRIP-seq signal coverage is shown to the right of the average profiles plot for one ORI SSR. B) As in A, but for SSRs not defined as origins (nonORI). B) As in A, but showing average DRIP-seq coverage for wild type cells (WT, purple) and *T. brucei* RNase H1 null mutants (green, *Tbrh1*-/-).

Figure S11. γH2A ChIP-seq signal is specifically enriched at sites of transcription initiation after TbRH2A depletion. Levels of γH2A ChIP-seq signal in TbRH2A RNAi induced sample coverage is plotted relative to uninduced sample coverage (each first normalised to input samples) for 24 hr (purple, low track) and 36 hr (green, upper track) time points in all 11 Mb-sized chromosomes. Scale: 1-3 fold-change. Other annotations are as in Fig. S5.

Figure S12. Gene ontology analysis reveals genes associated with antigenic variation are up-regulated after TbRH2A depletion, and those associated with small molecular biosynthesis pathways are down-regulated. Gene ontology analysis of biological process terms associated with genes found to be significantly up-regulated or down-regulated in RNA-seq analysis comparing RNA abundance after 36 hr TbRH2A of depletion via RNAi compared with uninduced cells. Parent terms are also depicted. Terms found to be enriched in the upregulated and downregulated genes are coloured green and orange, respectively. Colour intensity indicates significance, which was determined as adjusted p value. Terms with values > 0.01 were deemed not significant (white).

Figure S13. DRIP-seq signal is enriched across the BESs after TbRH2A depletion. DRIP-seq signal coverage (normalised to input coverage) is plotted across the BESs for WT (pink), TbRH2A uninduced (blue; tet -) and induced (orange, tet +) cells. The lowest track shows the structure of each BES; promoters (cyan), ESAGs (blue), 70-bp repeats (purple), VSGs (red) and other genes (green) are annotated as boxes. Black circles denote the end of the BES sequence assembly.

Figure S14. γH2A ChIP-seq is enriched across the BESs after TbRH2A depletion. Levels of γH2A ChIP-seq signal in TbRH2A RNAi induced sample coverage is plotted relative to uninduced sample

coverage (each first normalised to input samples) for 24 hr (purple, lower track) and 36 hr (green, upper track) time points, across each BESs. Scale, 1-3 fold-change. The lowest track shows the structure of each BES; promoters (cyan), ESAGs (blue), 70-bp repeats (purple), VSGs (red) and other genes (green) are annotated as boxes. Black circles denote the end of the BES sequence assembly.

Figure S15. DRIP-seq and γH2A ChIP-seq signal is enriched across rRNA genes after TbRH2A depletion. Upper diagrams shows DRIP-seq signal coverage (normalised to input coverage) plotted across the rRNA locus on chromosome 3 for TbRH2A uninduced (blue; tet -) andRNAi induced (orange, tet +) cells. CDS are indicated as black boxes. The lower diagrams shows the same rRNA locus but depicts levels of γH2A ChIP-seq signal, plotting coverage in TbRH2A RNAi induced samples relative to coverage in uninduced samples (each first normalised to input samples) for 24 hr (purple) and 36 hr (green) time points (scale, 1-3 fold-change)

Figure S16. Silent BES-housed VSGs are transcribed after TbRH2A depletion. RNA-seq read coverage is plotted over the coding regions of VSGs housed within silent BESs for two independent replicates (blue and orange) of TbRH2A RNAi parasites grown for 36 hr in the absence (tet -) or presence (tet +) of tet-induction. The VSG names and BESs in which they are contained are indicated.

Query ID	Hit Species	Hit Annotation	Accession number	E-value	Identity score
**Tb427.10.5070**	*Trypanosoma congolense*	Putative RNase H	CCC93661.1	0	539
	Trypanosoma cruzi	RNase H	XP_806655.1	4.00E-177	502
	Leishmania major	Putative RNase H	XP_001686636.1	1.00E-116	348
	Leishmania donovani	RNase H, putative	XP_003865186.1	2.00E-115	339
	Mus musculus	RNase H2 A subunit	NP_081463.1	3.00E-47	164
	Arabidopsis thaliana	Polynucleotidy transferase, ribonuclease-H like superfamily	NP_565584.1	3.00E-46	161
	Homo sapiens	RNase H2 A subunit	NP_006388.2	4.00E-45	159
	Drosophila melanogaster	Uncharacterised protein	NP_608521.1	7.00E-40	146
	Plasmodium falciparum	RNase H2 A subunit, putative	XP_966221.1	3.00E-40	145
	Saccharomyces cerevisiae	RNase H2 catalytic subunit RNH201	NP_014327.1	6.00E-40	145
	Caenorhabditis elegans	RNase H2 subunit A	NP_495796.1	4.00E-39	143
	Escherichia coli	RNase HII	NP_414725.1	1.00E-04	46.2
**Tb427.01.4220**	*Trypanosoma congolense*	conserved hypothetical protein	CCC89404.1	8.00E-143	421
	Trypanosoma cruzi	hypothetical protein	XP_808292.1	5.00E-103	322
	Danio rerio	ribonuclease H2 subunit B isoform X1	XP_021334305.1	7.00E-13	72.4
	Mus musculus	ribonuclease H2 subunit B	NP_080277.1	8.00E-04	45.1
	Homo sapiens	ribonuclease H2 subunit B isoform X3	XP_011533532.1	0.33	36.6
**Tb427.01.4730**	*Trypanosoma brucei equiperdum*	Ribonuclease H2 non-catalytic subunit	RHW74391.1	8.00E-113	326
	Trypanosoma congolense	unnamed protein product	CCD13112.1	1.00E-50	168
	Trypanosoma cruzi	hypothetical protein	XP_809724.1	2.00E-43	149
	Leishmania braziliensis	Ribonuclease_H2_non-catalytic_subunit	SYZ63788.1	7.00E-19	87.8
	Daphnia magna	Ribonuclease H2 subunit C	KZS21140.1	0.27	41.2

Table. S1

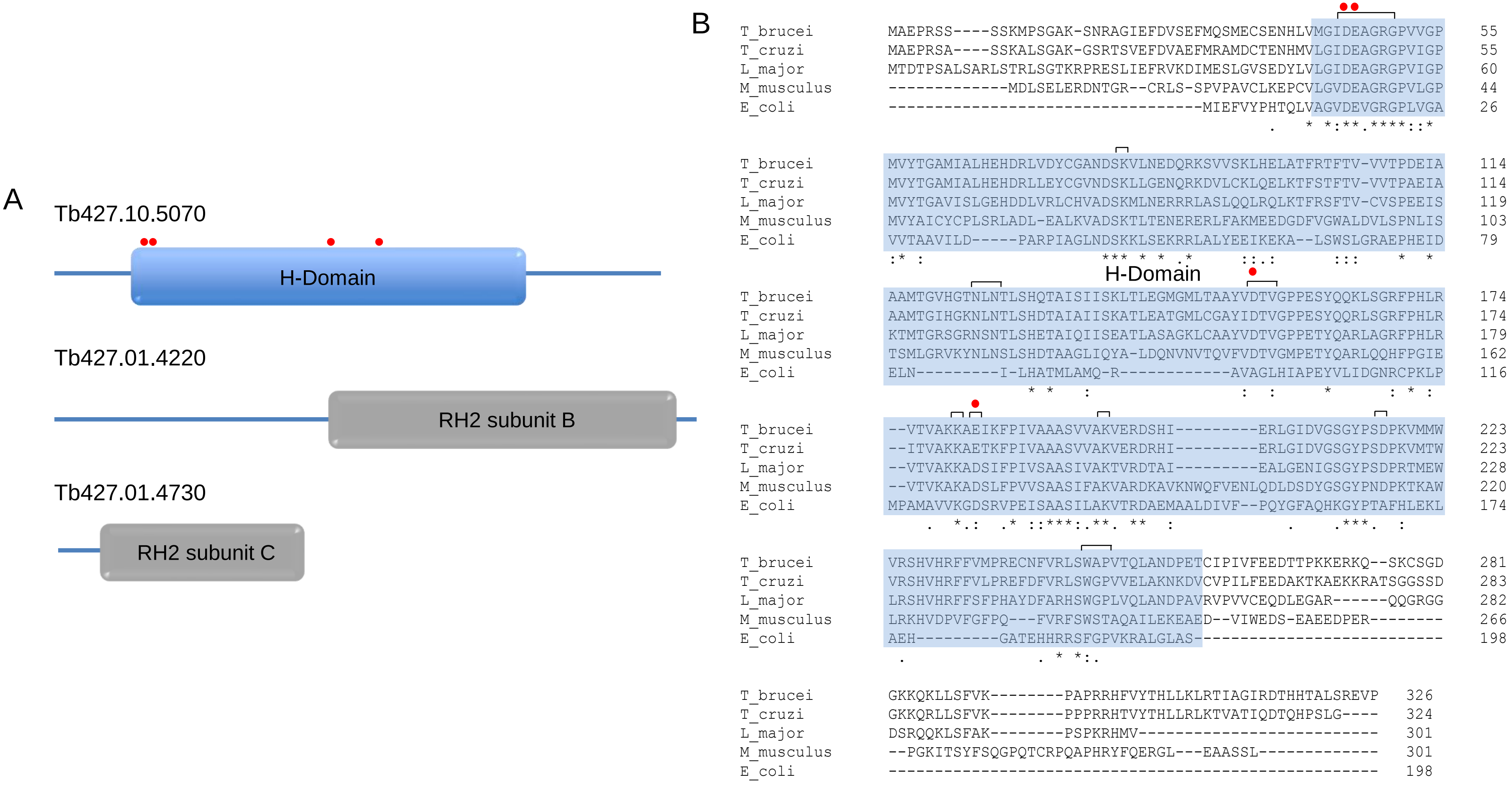
A
Tb427.10.5070
H-Domain
Tb427.01.4220
RH2 subunit B
Tb427.01.4730
RH2 subunit C
B
T_brucei MAEPRSS----SSKMPSGAK-SNRAGIEFDVSEFMQSMECSENHLVMGIDEAGRGPVVGP 55
T_cruzi MAEPRSA----SSKALSGAK-GSRTSVEFDVAEFMRAMDCTENHMVLGIDEAGRGPVIGP 55
L_major MTDTPSALSARLSTRLSGTKRPRESLIEFRVKDIMESLGVSEDYLVLGIDEAGRGPVIGP 60
M_musculus -------------MDLSELERDNTGR--CRLS-SPVPAVCLKEPCVLGVDEAGRGPVLGP 44
E_coli ----------------------------------MIEFVYPHTQLVAGVDEVGRGPLVGA 26
. * *:**.****::*
T_brucei MVYTGAMIALHEHDRLVDYCGANDSKVLNEDQRKSVVSKLHELATFRTFTV-VVTPDEIA 114
T_cruzi MVYTGAMIALHEHDRLLEYCGVNDSKLLGENQRKDVLCKLQELKTFSTFTV-VVTPAEIA 114
L_major MVYTGAVISLGEHDDLVRLCHVADSKMLNERRRLASLQQLRQLKTFRSFTV-CVSPEEIS 119
M_musculus MVYAICYCPLSRLADL-EALKVADSKTLTENERERLFAKMEEDGDFVGWALDVLSPNLIS 103
E_coli VVTAAVILD-----PARPIAGLNDSKKLSEKRRLALYEEIKEKA--LSWSLGRAEPHEID 79
:* : *** * * .* ::.: ::: * *
H-Domain
T_brucei AAMTGVHGTNLNTLSHQTAISIISKLTLEGMGMLTAAYVDTVGPPESYQQKLSGRFPHLR 174
T_cruzi AAMTGIHGKNLNTLSHDTAIAIISKATLEATGMLCGAYIDTVGPPESYQQRLSGRFPHLR 174
L_major KTMTGRSGRNSNTLSHETAIQIISEATLASAGKLCAAYVDTVGPPETYQARLAGRFPHLR 179
M_musculus TSMLGRVKYNLNSLSHDTAAGLIQYA-LDQNVNVTQVFVDTVGMPETYQARLQQHFPGIE 162
E_coli ELN---------I-LHATMLAMQ-R------------AVAGLHIAPEYVLIDGNRCPKLP 116
* * : : : * : * :
T_brucei --VTVAKKAEIKFPIVAAASVVAKVERDSHI---------ERLGIDVGSGYPSDPKVMMW 223
T_cruzi --ITVAKKAETKFPIVAAASVVAKVERDRHI---------ERLGIDVGSGYPSDPKVMTW 223
L_major --VTVAKKADSIFPIVSAASIVAKTVRDTAI---------EALGENIGSGYPSDPRTMEW 228
M_musculus --VTVKAKADSLFPVVSAASIFAKVARDKAVKNWQFVENLQDLDSDYGSGYPNDPKTKAW 220
E_coli MPAMAVVKGDSRVPEISAASILAKVTRDAEMAALDIVF--PQYGFAQHKGYPTAFHLEKL 174
. *.: .* ::***:.**. ** : . .***. :
T_brucei VRSHVHRFFVMPRECNFVRLSWAPVTQLANDPETCIPIVFEEDTTPKKERKQ--SKCSGD 281
T_cruzi VRSHVHRFFVLPREFDFVRLSWGPVVELAKNKDVCVPILFEEDAKTKAEKKRATSGGSSD 283
L_major LRSHVHRFFSFPHAYDFARHSWGPLVQLANDPAVRVPVVCEQDLEGAR------QQGRGG 282
M_musculus LRKHVDPVFGFPQ---FVRFSWSTAQAILEKEAED--VIWEDS-EAEEDPER-------- 266
E_coli AEH---------GATEHHRRSFGPVKRALGLAS--------------------------- 198
. . * *:.
T_brucei GKKQKLLSFVK--------PAPRRHFVYTHLLKLRTIAGIRDTHHTALSREVP 326
T_cruzi GKKQRLLSFVK--------PPPRRHTVYTHLLRLKTVATIQDTQHPSLG---- 324
L_major DSRQQKLSFAK--------PSPKRHMV-------------------------- 301
M_musculus --PGKITSYFSQGPQTCRPQAPHRYFQERGL---EAASSL------------- 301
E_coli --- 198

Fig. S1

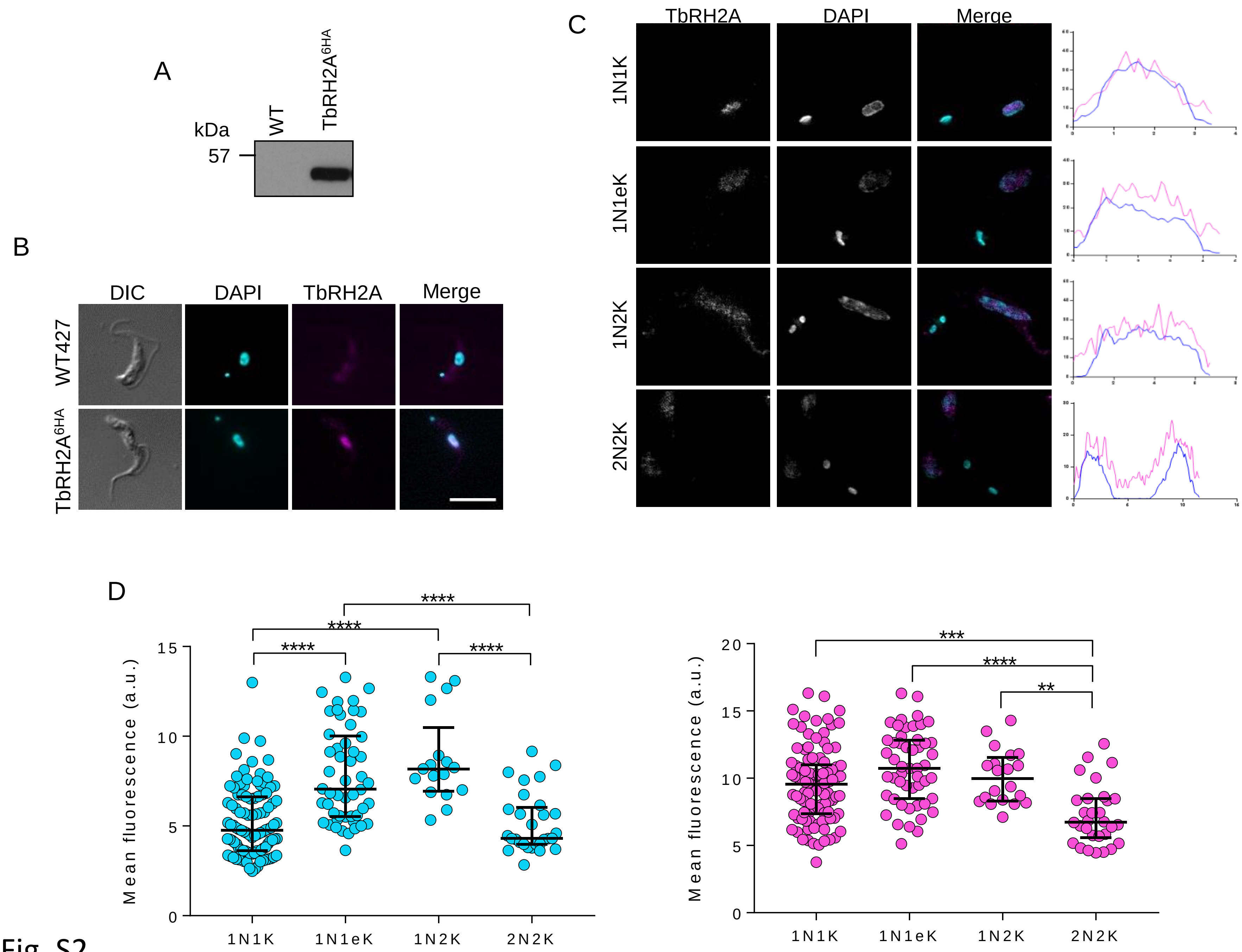
A
kDa
57
WT
TbRH2A6HA
B
DIC
DAPI
TbRH2A
Merge
WT427
TbRH2A6HA
C
TbRH2A
DAPI
Merge
1N1K
1N1eK
1N2K
2N2K
D
Mean fluorescence (a.u.)
1N1K
1N1eK
1N2K
2N2K
****
***
**

Fig. S2

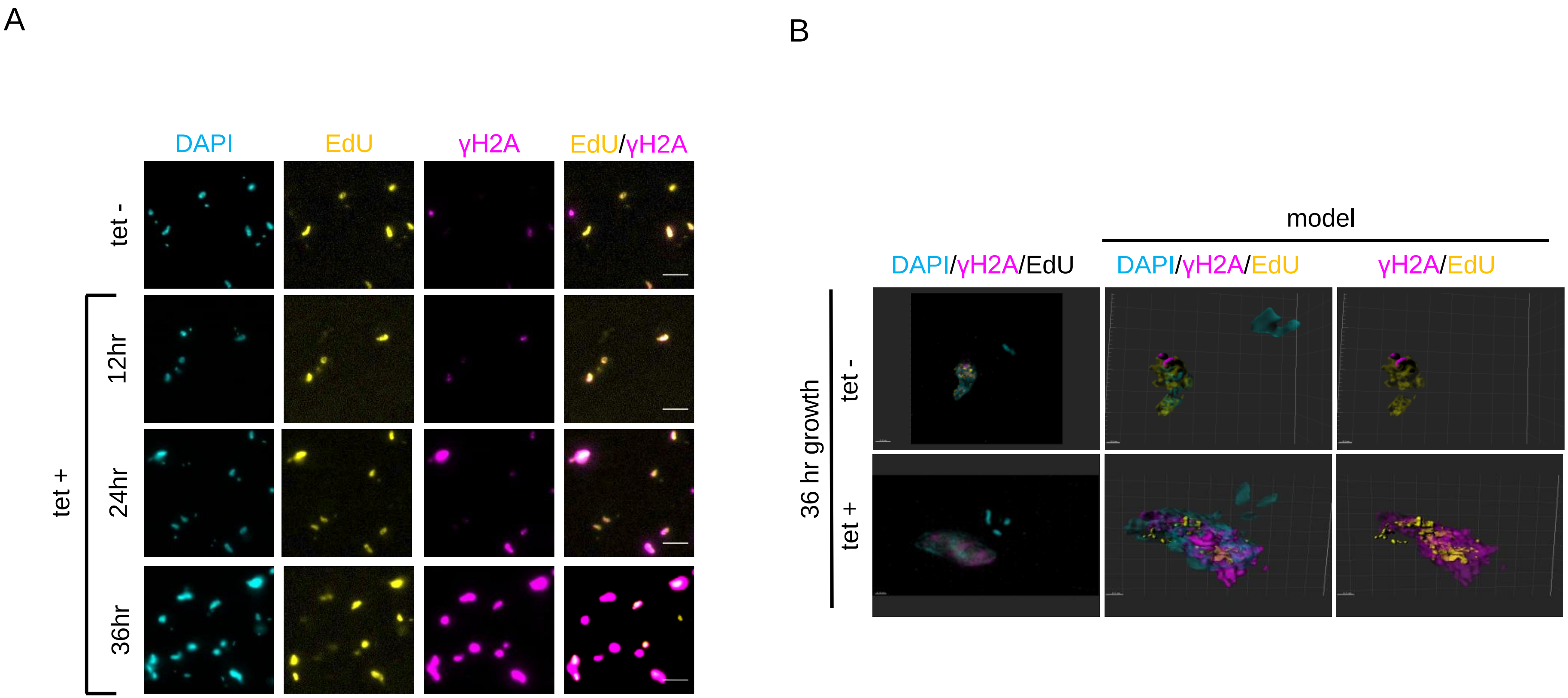
A
DAPI
EdU
γH2A
EdU/γH2A
tet -
tet +
12hr
24hr
36hr
B
model
DAPI/γH2A/EdU
DAPI/γH2A/EdU
γH2A/EdU
36 hr growth
tet -
tet +

Fig. S3

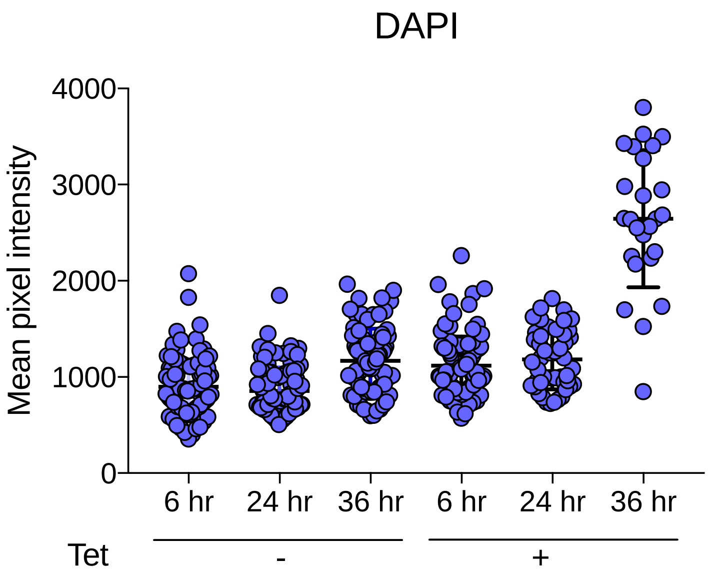
DAPI
Mean pixel intensity
4000
3000
2000
1000
0
6 hr
24 hr
36 hr
6 hr
24 hr
36 hr
Tet
-
+

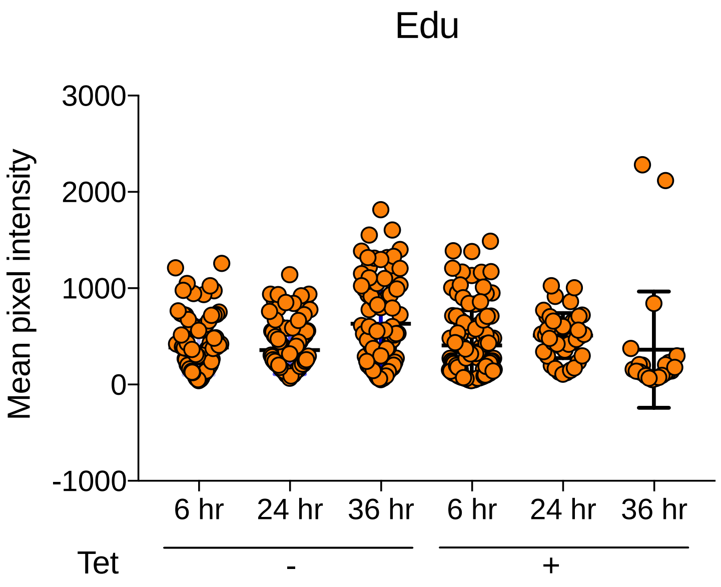
Edu
Mean pixel intensity
3000
2000
1000
0
-1000
6 hr
24 hr
36 hr
6 hr
24 hr
36 hr
Tet
-
+

Fig. S4

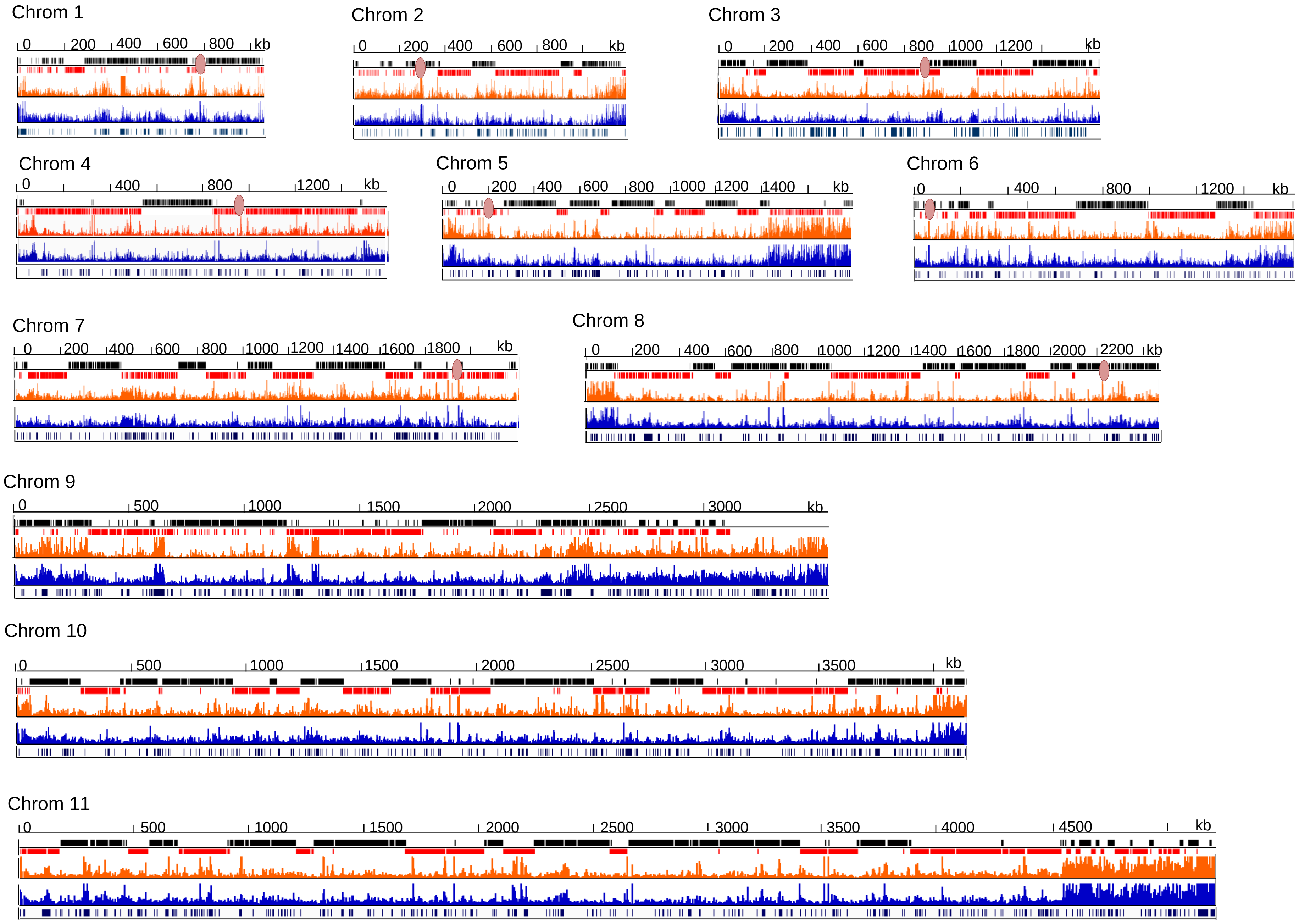
Chrom 1
0 200 400 600 800 kb
Chrom 2
0 200 400 600 800 kb
Chrom 3
0 200 400 600 800 1000 1200 kb
Chrom 4
0 400 800 1200 kb
Chrom 5
0 200 400 600 800 1000 1200 1400 kb
Chrom 6
0 400 800 1200 kb
Chrom 7
0 200 400 600 800 1000 1200 1400 1600 1800 kb
Chrom 8
0 200 400 600 800 1000 1200 1400 1600 1800 2000 2200 kb
Chrom 9
0 500 1000 1500 2000 2500 3000 kb
Chrom 10
0 500 1000 1500 2000 2500 3000 3500 kb
Chrom 11
0 500 1000 1500 2000 2500 3000 3500 4000 4500 kb

Fig. S5

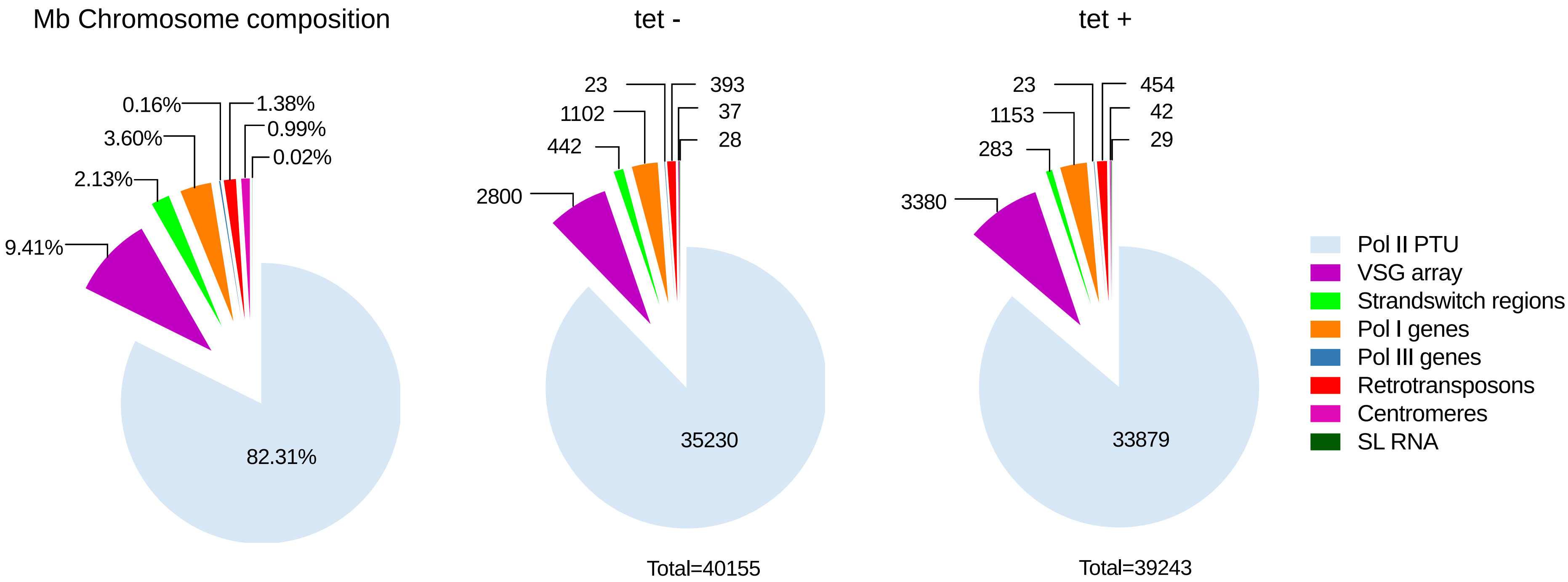
Mb Chromosome composition
tet -
tet +
0.16%
1.38%
0.99%
3.60%
0.02%
2.13%
9.41%
82.31%
23
393
1102
37
442
28
2800
35230
Total=40155
23
454
1153
42
283
29
3380
33879
Total=39243
Pol II PTU
VSG array
Strandswitch regions
Pol I genes
Pol III genes
Retrotransposons
Centromeres
SL RNA

Fig. S6

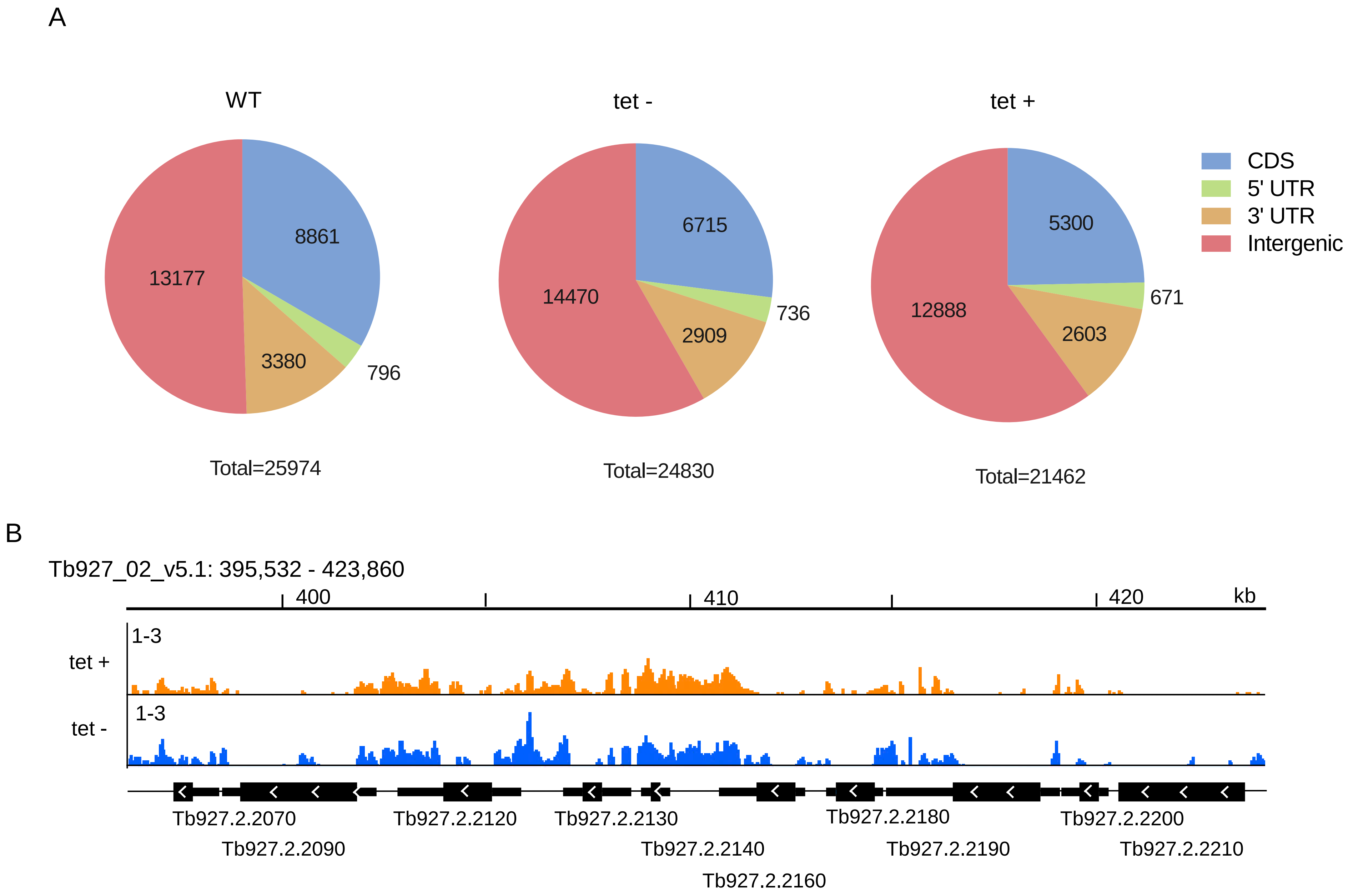
A
WT
tet -
tet +
8861
13177
3380
796
6715
14470
2909
736
5300
12888
2603
671
CDS
5' UTR
3' UTR
Intergenic
Total=25974
Total=24830
Total=21462
B
Tb927_02_v5.1: 395,532 - 423,860
400
410
420
kb
tet +
1-3
tet -
1-3
Tb927.2.2070
Tb927.2.2090
Tb927.2.2120
Tb927.2.2130
Tb927.2.2140
Tb927.2.2160
Tb927.2.2180
Tb927.2.2190
Tb927.2.2200
Tb927.2.2210

Fig. S7

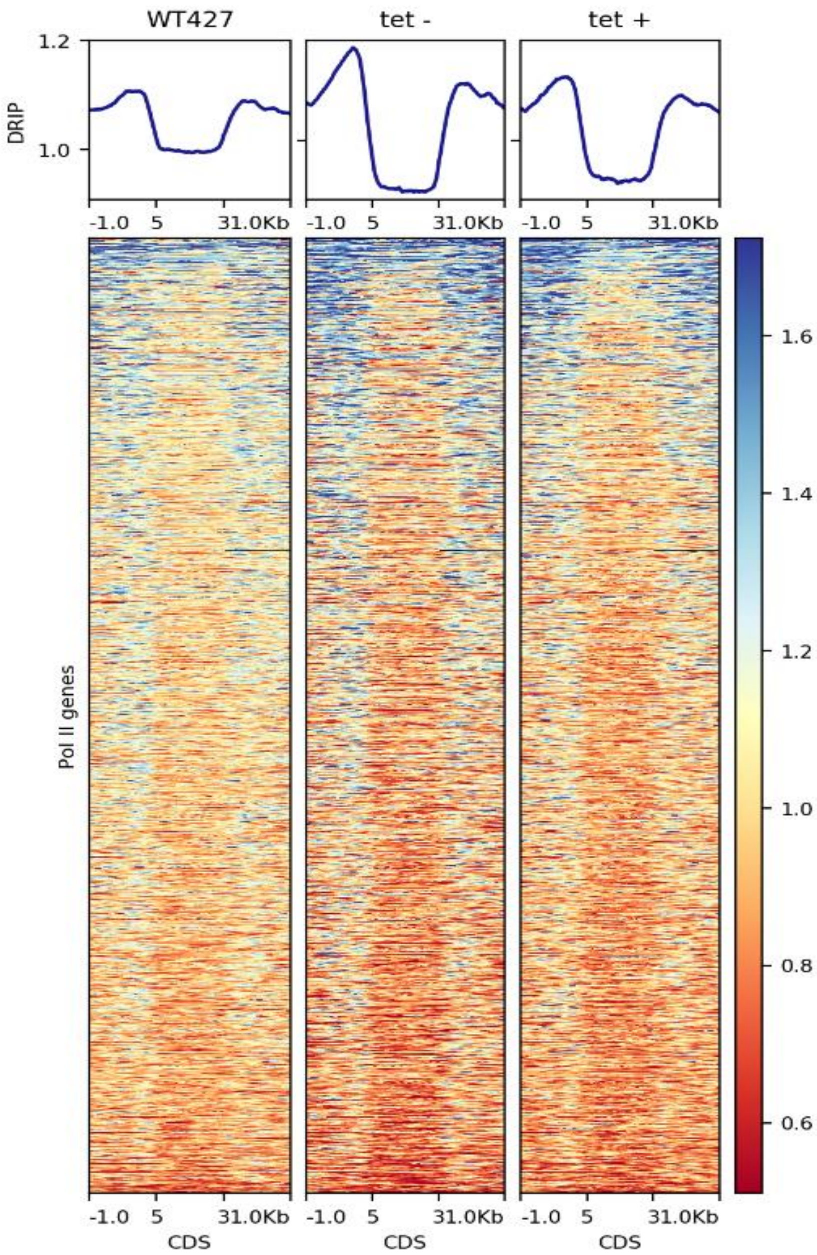
WT427
tet -
tet +
DRIP
1.2
1.0
-1.0
5
31.0Kb
1.6
1.4
1.2
1.0
0.8
0.6
Pol II genes
CDS

Fig. S8

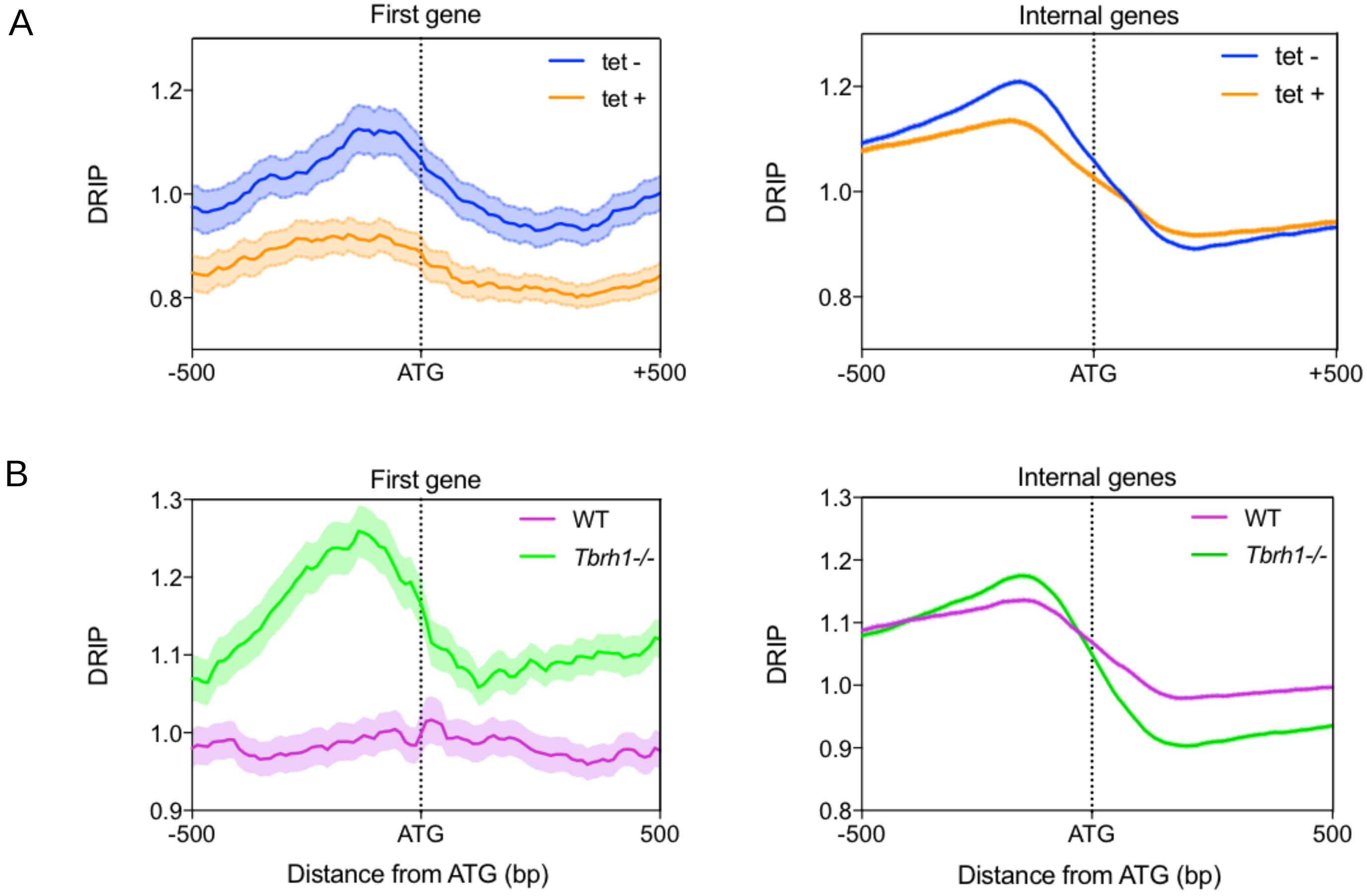
A
First gene
tet -
tet +
1.2
1.0
0.8
DRIP
-500
ATG
+500
Internal genes
tet -
tet +
1.2
1.0
0.8
DRIP
-500
ATG
+500
B
First gene
WT
Tbrh1-/-
1.3
1.2
1.1
1.0
0.9
DRIP
-500
ATG
500
Distance from ATG (bp)
Internal genes
WT
Tbrh1-/-
1.3
1.2
1.1
1.0
0.9
0.8
DRIP
-500
ATG
500
Distance from ATG (bp)

Fig. S9

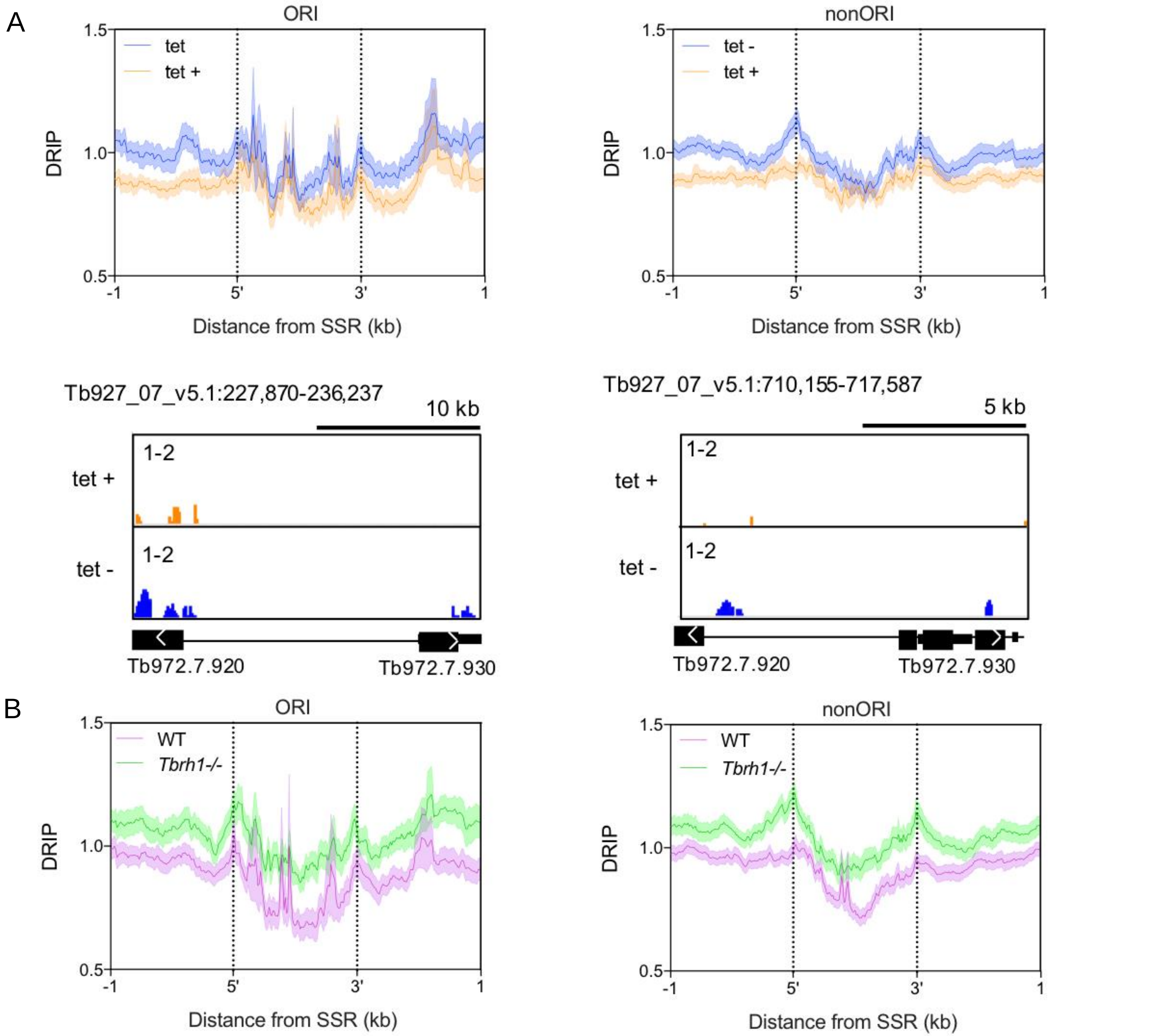
A
ORI
tet
tet +
DRIP
1.5
1.0
0.5
-1
5'
3'
1
Distance from SSR (kb)
nonORI
tet -
tet +
Tb927_07_v5.1:227,870-236,237
10 kb
tet +
1-2
tet -
1-2
Tb972.7.920
Tb972.7.930
Tb927_07_v5.1:710,155-717,587
5 kb
B
WT
Tbrh1-/-

Fig. S10

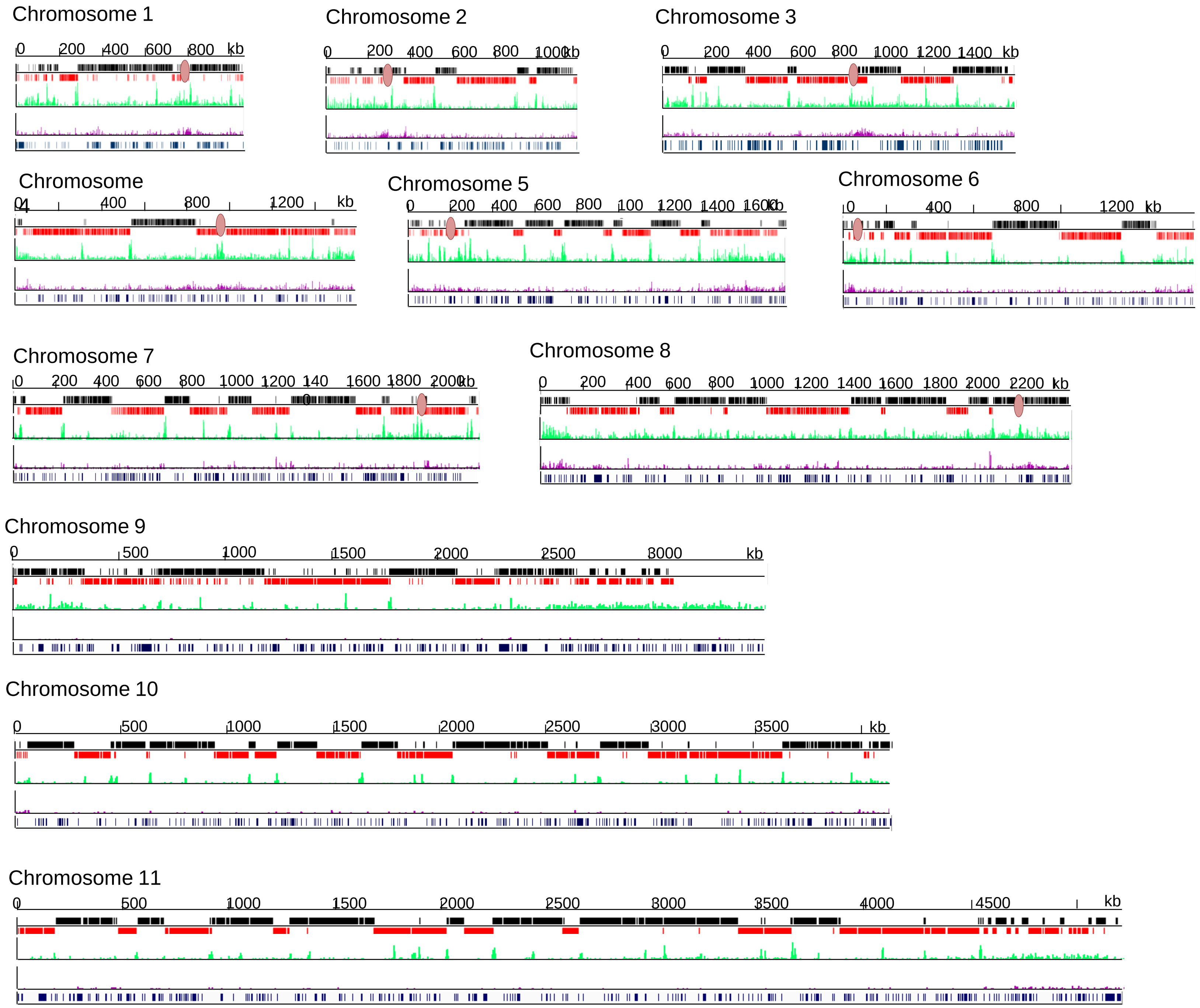
Chromosome 1
0 200 400 600 800 kb
Chromosome 2
0 200 400 600 800 1000 kb
Chromosome 3
0 200 400 600 800 1000 1200 1400 kb
Chromosome 4
0 400 800 1200 kb
Chromosome 5
0 200 400 600 800 100 1200 1400 1600 kb
Chromosome 6
0 400 800 1200 kb
Chromosome 7
0 200 400 600 800 1000 1200 140 1600 1800 2000 kb
Chromosome 8
0 200 400 600 800 1000 1200 1400 1600 1800 2000 2200 kb
Chromosome 9
0 500 1000 1500 2000 2500 3000 kb
Chromosome 10
0 500 1000 1500 2000 2500 3000 3500 kb
Chromosome 11
0 500 1000 1500 2000 2500 3000 3500 4000 4500 kb

Fig. S11

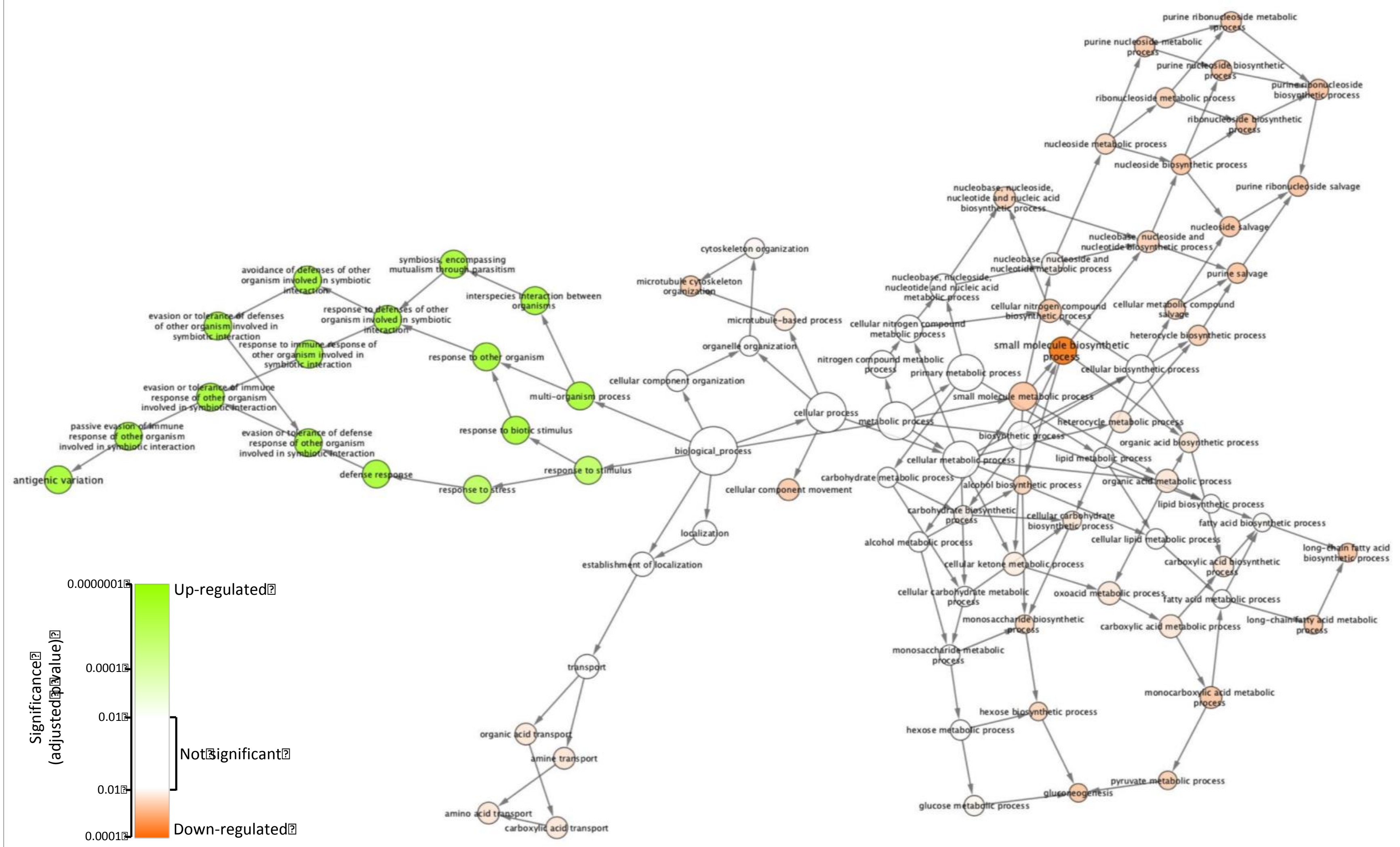

Up-regulated
Significance (adjusted p-value)
0.0000001
0.0001
0.01
0.01
0.0001
Not significant
Down-regulated
antigenic variation
passive evasion of immune response of other organism involved in symbiotic interaction
evasion or tolerance of immune response of other organism involved in symbiotic interaction
evasion or tolerance of defenses of other organism involved in symbiotic interaction
avoidance of defenses of other organism involved in symbiotic interaction
response to immune response of other organism involved in symbiotic interaction
response to defenses of other organism involved in symbiotic interaction
evasion or tolerance of defense response of other organism involved in symbiotic interaction
symbiosis, encompassing mutualism through parasitism
interspecies interaction between organisms
response to other organism
multi-organism process
response to biotic stimulus
defense response
response to stress
response to stimulus
biological_process
cellular component organization
cytoskeleton organization
microtubule cytoskeleton organization
microtubule-based process
organelle organization
cellular process
cellular component movement
localization
establishment of localization
transport
organic acid transport
amine transport
amino acid transport
carboxylic acid transport
metabolic process
primary metabolic process
nitrogen compound metabolic process
cellular nitrogen compound metabolic process
nucleobase, nucleoside, nucleotide and nucleic acid metabolic process
nucleobase, nucleoside, nucleotide and nucleic acid biosynthetic process
nucleobase, nucleoside and nucleotide metabolic process
nucleobase, nucleoside and nucleotide biosynthetic process
cellular nitrogen compound biosynthetic process
small molecule biosynthetic process
small molecule metabolic process
cellular biosynthetic process
biosynthetic process
heterocycle metabolic process
heterocycle biosynthetic process
cellular metabolic compound salvage
purine salvage
nucleoside salvage
purine ribonucleoside salvage
nucleoside metabolic process
nucleoside biosynthetic process
ribonucleoside metabolic process
ribonucleoside biosynthetic process
purine nucleoside metabolic process
purine nucleoside biosynthetic process
purine ribonucleoside metabolic process
purine ribonucleoside biosynthetic process
cellular metabolic process
carbohydrate metabolic process
carbohydrate biosynthetic process
alcohol biosynthetic process
alcohol metabolic process
cellular carbohydrate biosynthetic process
cellular carbohydrate metabolic process
cellular ketone metabolic process
monosaccharide biosynthetic process
monosaccharide metabolic process
hexose biosynthetic process
hexose metabolic process
glucose metabolic process
gluconeogenesis
lipid metabolic process
organic acid metabolic process
organic acid biosynthetic process
lipid biosynthetic process
fatty acid biosynthetic process
long-chain fatty acid biosynthetic process
cellular lipid metabolic process
carboxylic acid biosynthetic process
oxoacid metabolic process
fatty acid metabolic process
long-chain fatty acid metabolic process
carboxylic acid metabolic process
monocarboxylic acid metabolic process
pyruvate metabolic process

Fig. S12

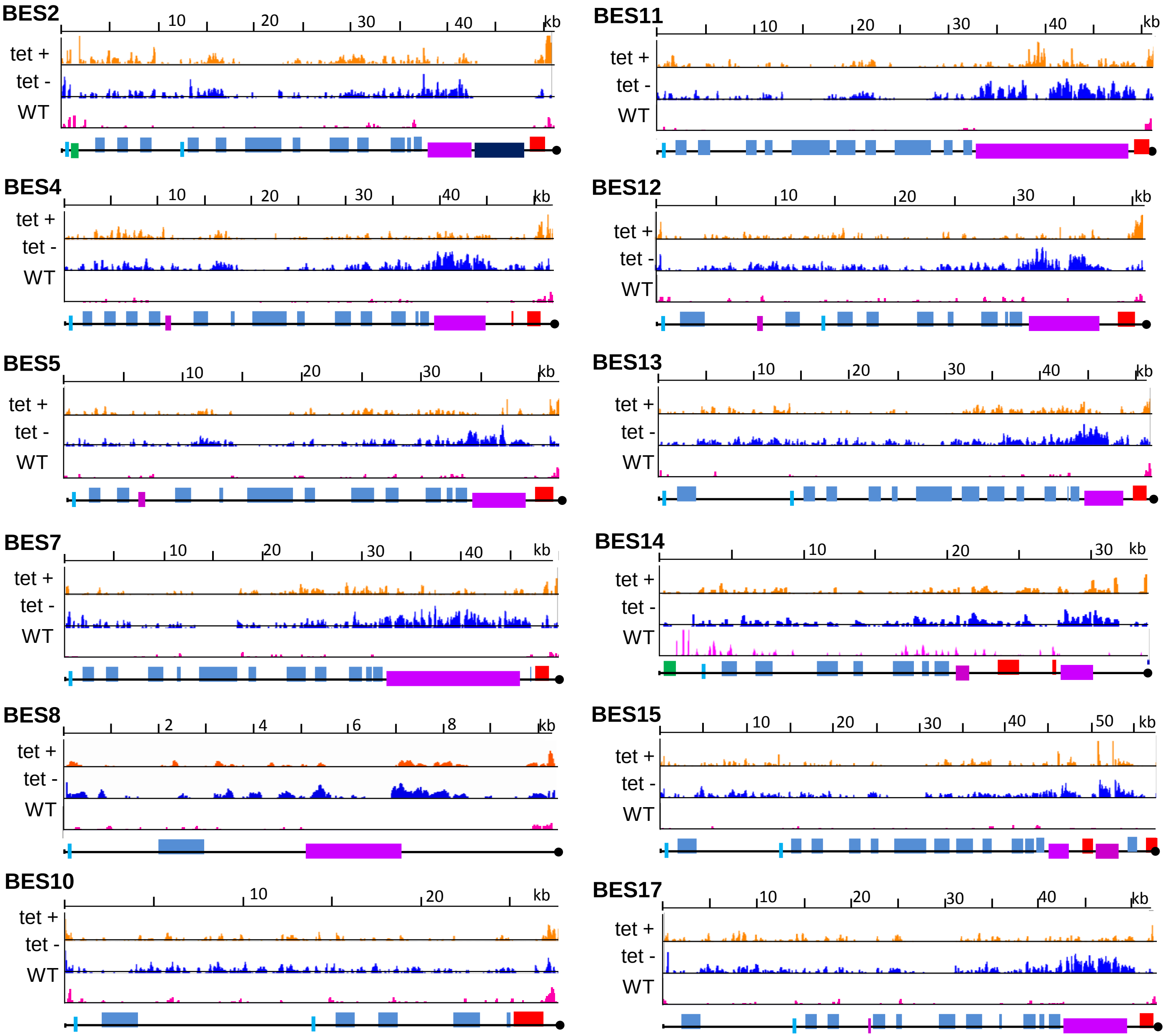
BES2
BES4
BES5
BES7
BES8
BES10
BES11
BES12
BES13
BES14
BES15
BES17
tet +
tet -
WT
kb
10
20
30
40
50
2
4
6
8

Fig. S13

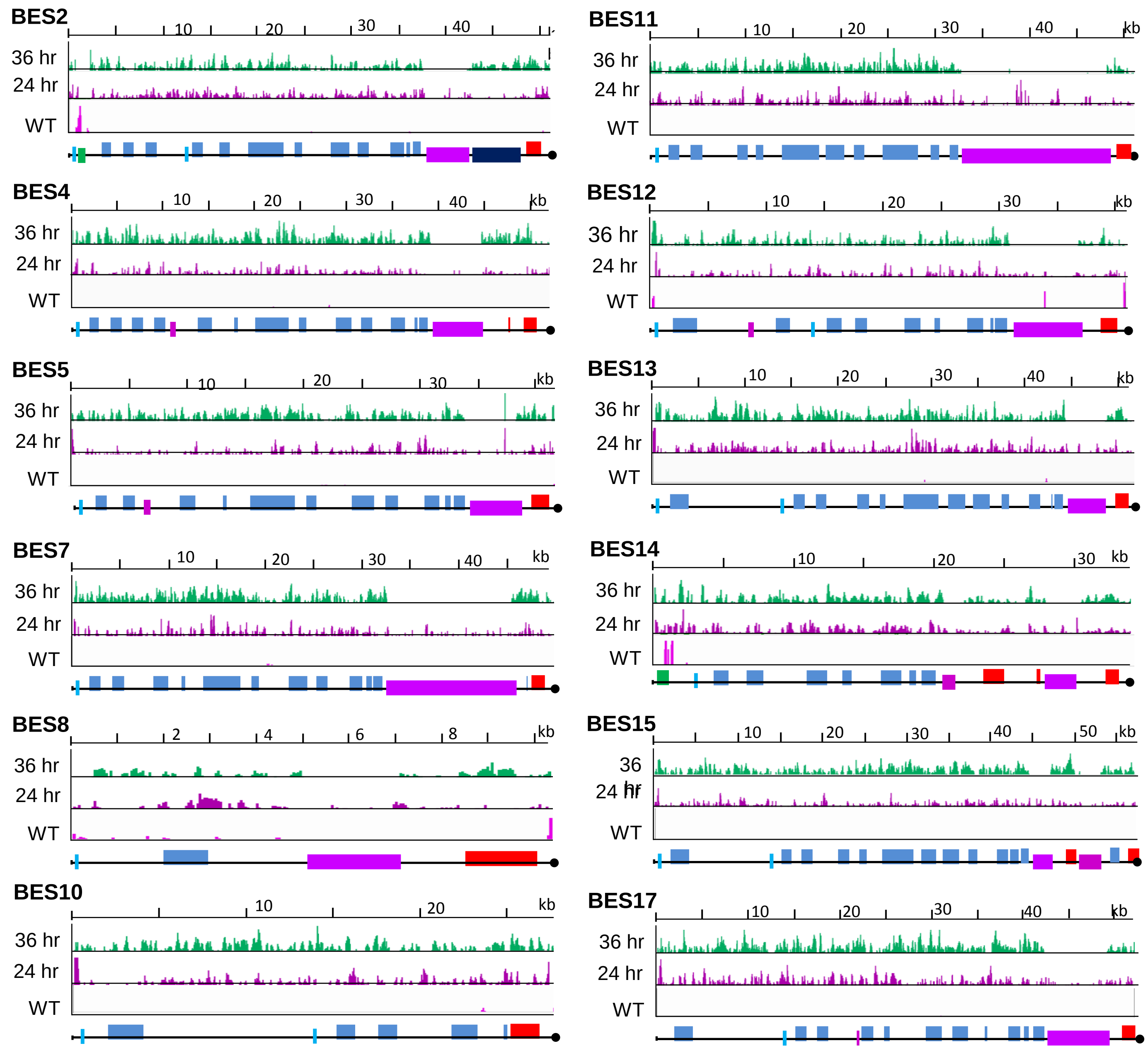
BES2
BES11
BES4
BES12
BES5
BES13
BES7
BES14
BES8
BES15
BES10
BES17
36 hr
24 hr
WT
kb

Fig. S14

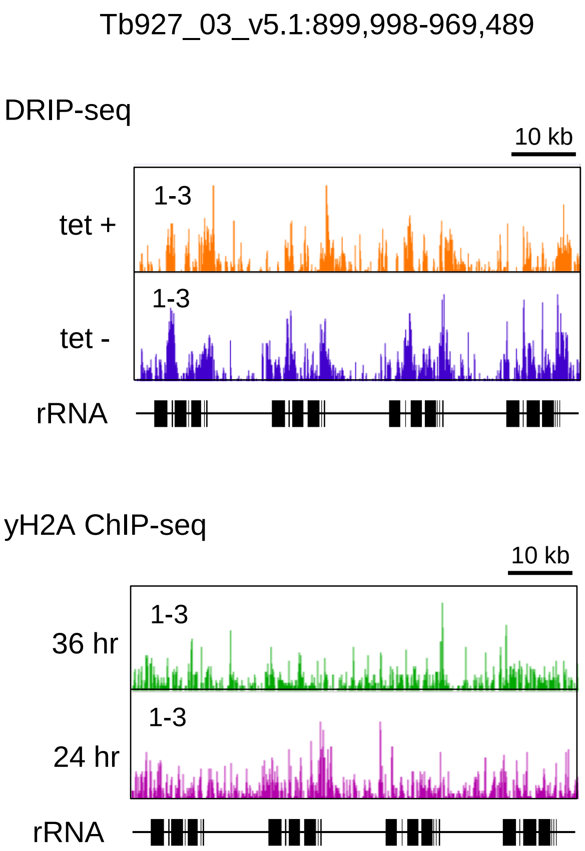

Tb927_03_v5.1:899,998-969,489
DRIP-seq
10 kb
1-3
tet +
1-3
tet -
rRNA
yH2A ChIP-seq
10 kb
1-3
36 hr
1-3
24 hr
rRNA

Fig. S15

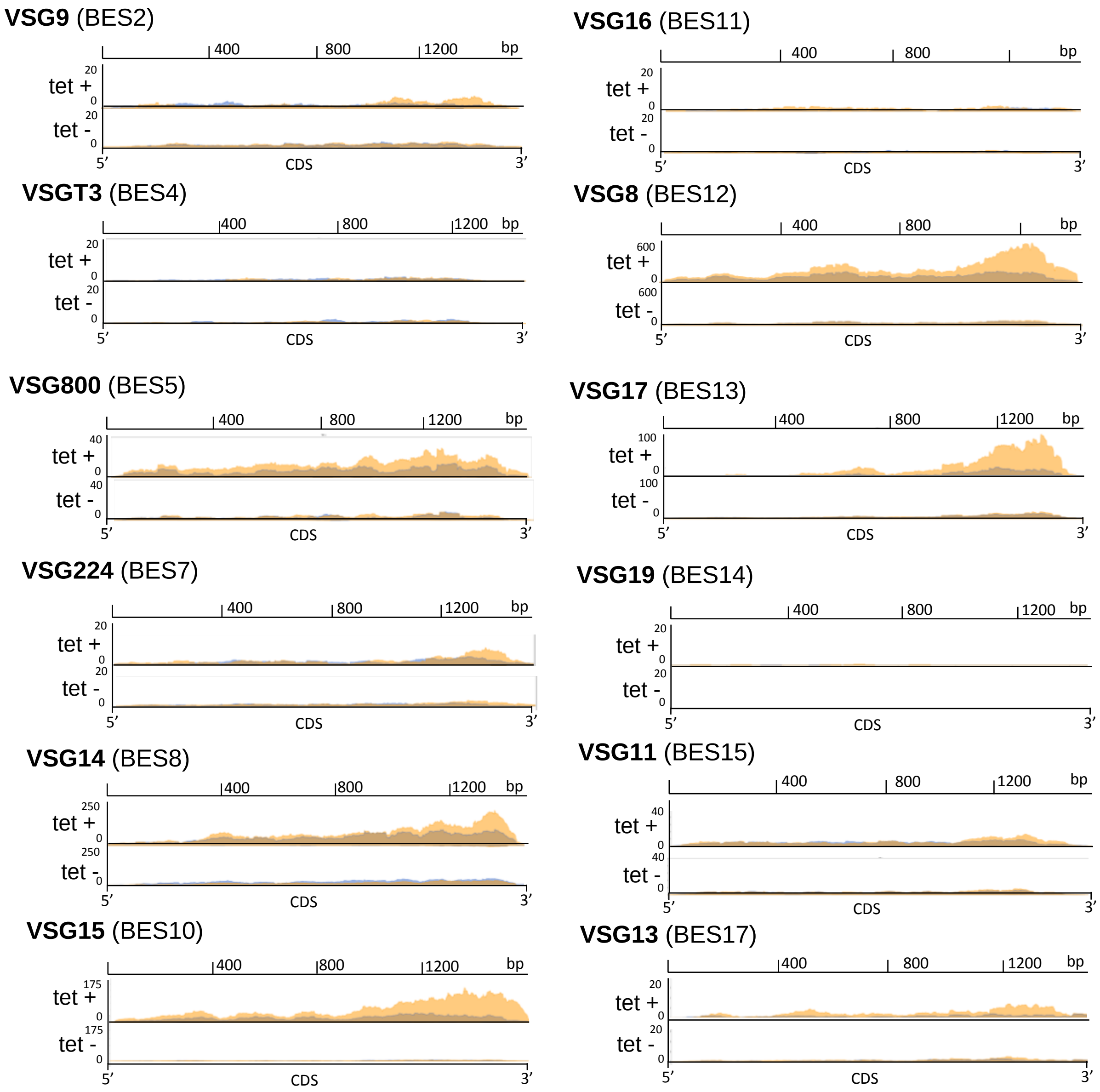
VSG9 (BES2)
VSG16 (BES11)
VSGT3 (BES4)
VSG8 (BES12)
VSG800 (BES5)
VSG17 (BES13)
VSG224 (BES7)
VSG19 (BES14)
VSG14 (BES8)
VSG11 (BES15)
VSG15 (BES10)
VSG13 (BES17)
400
800
1200
bp
tet +
tet -
5′
CDS
3′

Fig. S16
